# Supplementary material for: Comprehensive Genetic Characterization of Mitochondrial Ca2+ Uniporter Components Reveals Their Different Physiological Requirements In Vivo
Source: Cell Rep. 2019 Apr 30;27(5):1541–1550.e5. doi: 10.1016/j.celrep.2019.04.033 (PMC6506686; doi:10.1016/j.celrep.2019.04.033)
Supplement: Document S1. Figures S1–S5 and Table S1 [file mmc1.pdf]

Cell Reports, Volume *27*

Supplemental Information

Comprehensive Genetic Characterization of Mitochondrial Ca^{2+} Uniporter Components Reveals Their Different Physiological Requirements *In Vivo*

Roberta Tufi, Thomas P. Gleeson, Sophia von Stockum, Victoria L. Hewitt, Juliette J. Lee, Ana Terriente-Felix, Alvaro Sanchez-Martinez, Elena Ziviani, and Alexander J. Whitworth

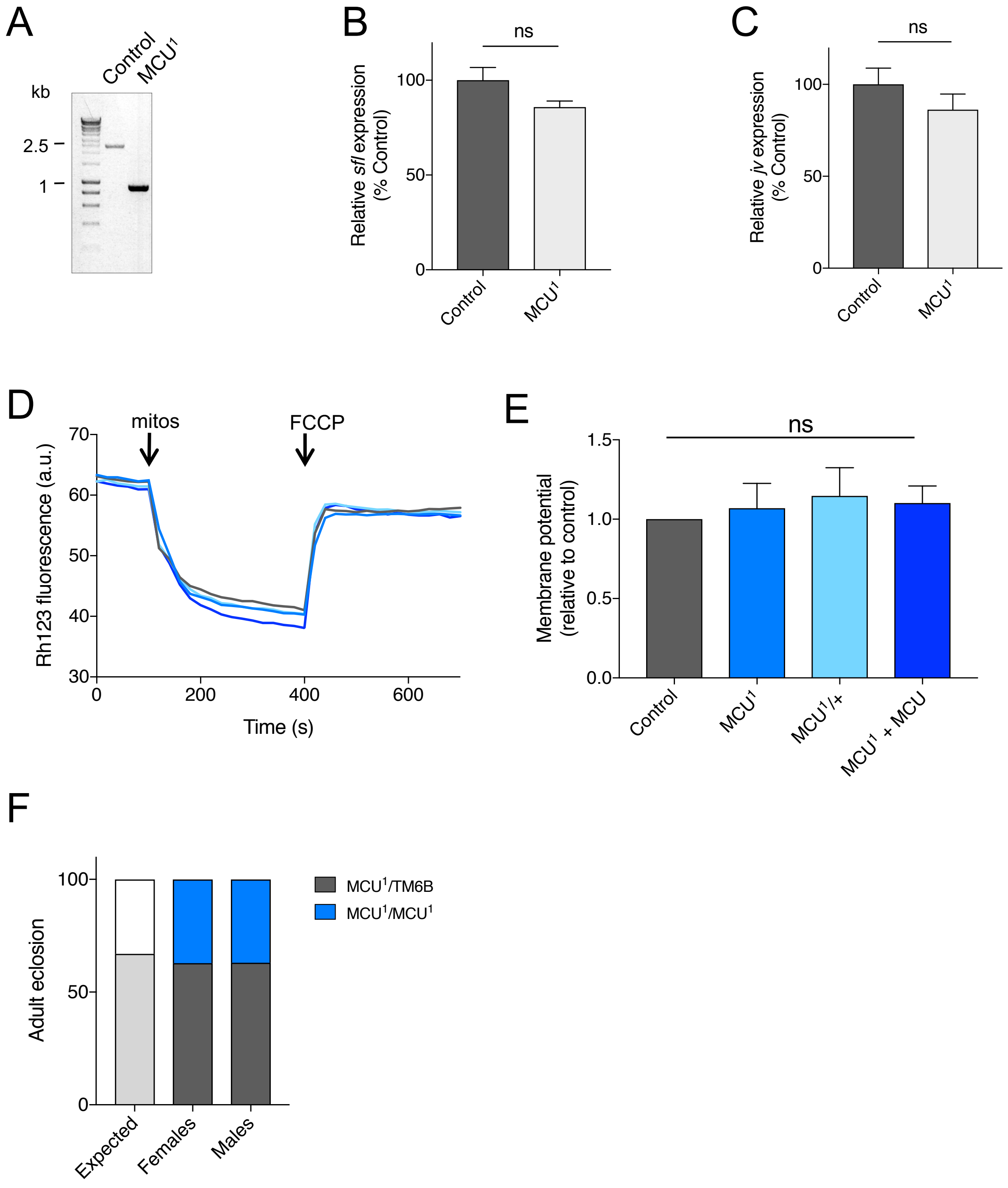

Figure S1. Additional characterisation of *MCU*¹ mutants. Related to Figure 1.
A. DNA analysis of *MCU*¹. Primer sequences are detailed in the 'STAR Methods' section. The control genotype yielded a ~2.5 kb band, compared to ~900 bp for *MCU*¹ homozygotes.
B, C. Relative transcript level of *sfl* (**B**) or *jv* (**C**) for control and *MCU*¹ flies (mean ± SD; n = 3).
D. Representative traces of Rhodamine 123 (Rh123) fluorescence (a.u.: arbitrary units) in isolated mitochondria of the indicated genotypes used for the Ca^{2+} uptake assays for *MCU* (with *da-GAL4* expressed *UAS-MCU*).
E. Relative membrane potential was determined through Rh123 fluorescence change before and after addition of FCCP (ΔF-F0) normalized to controls (mean ± SEM; n = 3).
F. The percentage of adult flies eclosing as homozygous *MCU*¹ mutants *versus* balanced heterozygotes, together with the expected Mendelian ratio in the offspring (n > 700).
The control genotype is w^{1118}.

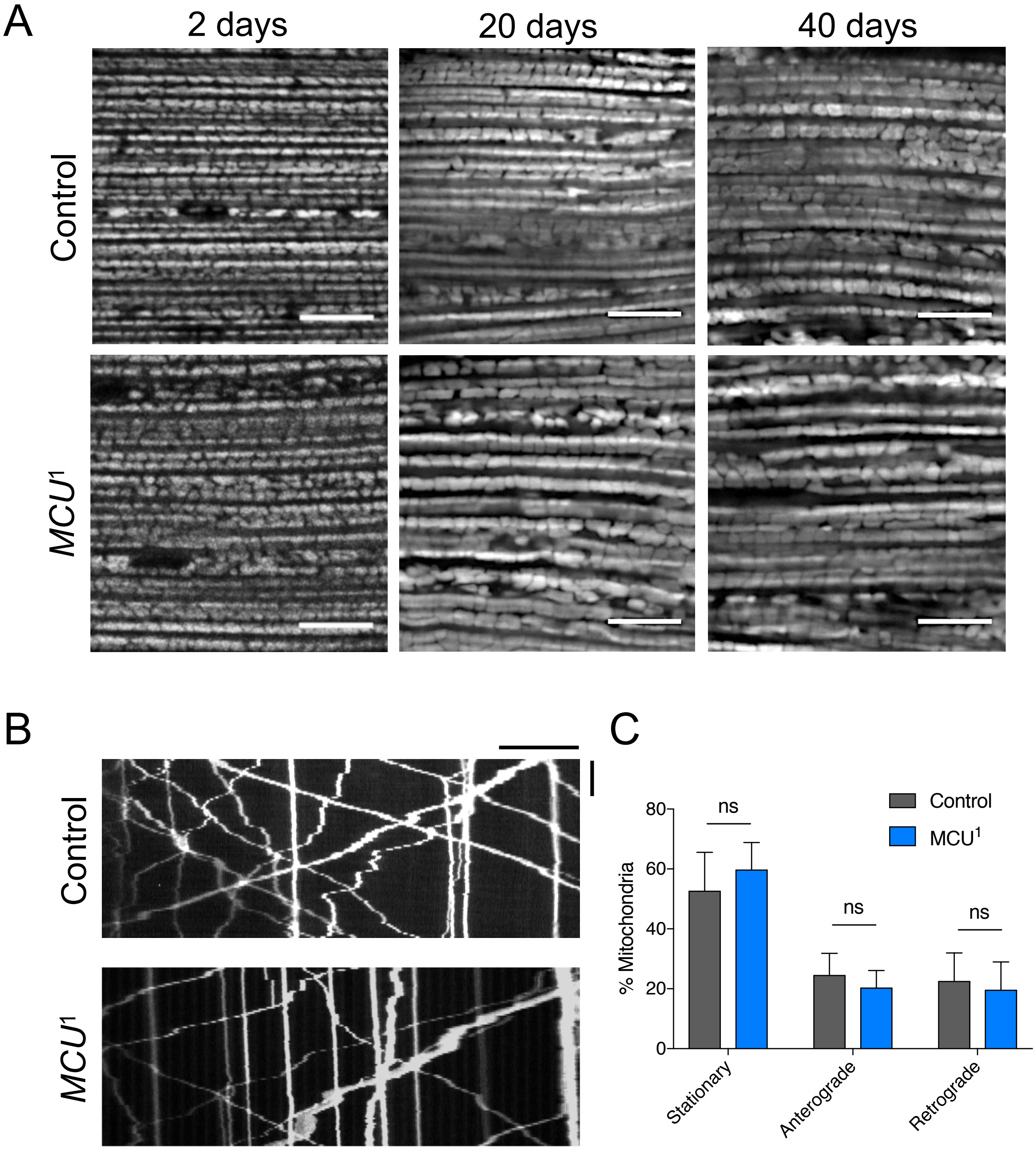

Figure S2. Mitochondrial morphology and dynamics in *MCU*¹ mutants. Related to Figure 1.
A. Mitochondrial morphology of control or *MCU*¹ adult indirect flight muscle at 2, 20, or 40 days post-eclosion. Scale bar = 10 µm. Genotypes - Control: *UAS-mito-HA-GFP/+; da-GAL4/+*. *MCU¹*: *UAS-mito-HA-GFP/+; MCU¹/MCU¹, da-GAL4*.
B. Representative kymographs of mitochondrial axonal transport in control and *MCU*¹ larvae. Scale bars: horizontal = 20 µm, vertical = 100 s. Genotypes - Control: *CCAP-GAL4, UAS-mito.tdTomato/+*. *MCU¹*: *CCAP-GAL4, UAS-mito.tdTomato/+; MCU¹/MCU¹*.
C. Quantification of mitochondrial transport shown in B. Statistical analysis: one-way ANOVA (mean ± 95% CI; n = 10; ns, non-significant).

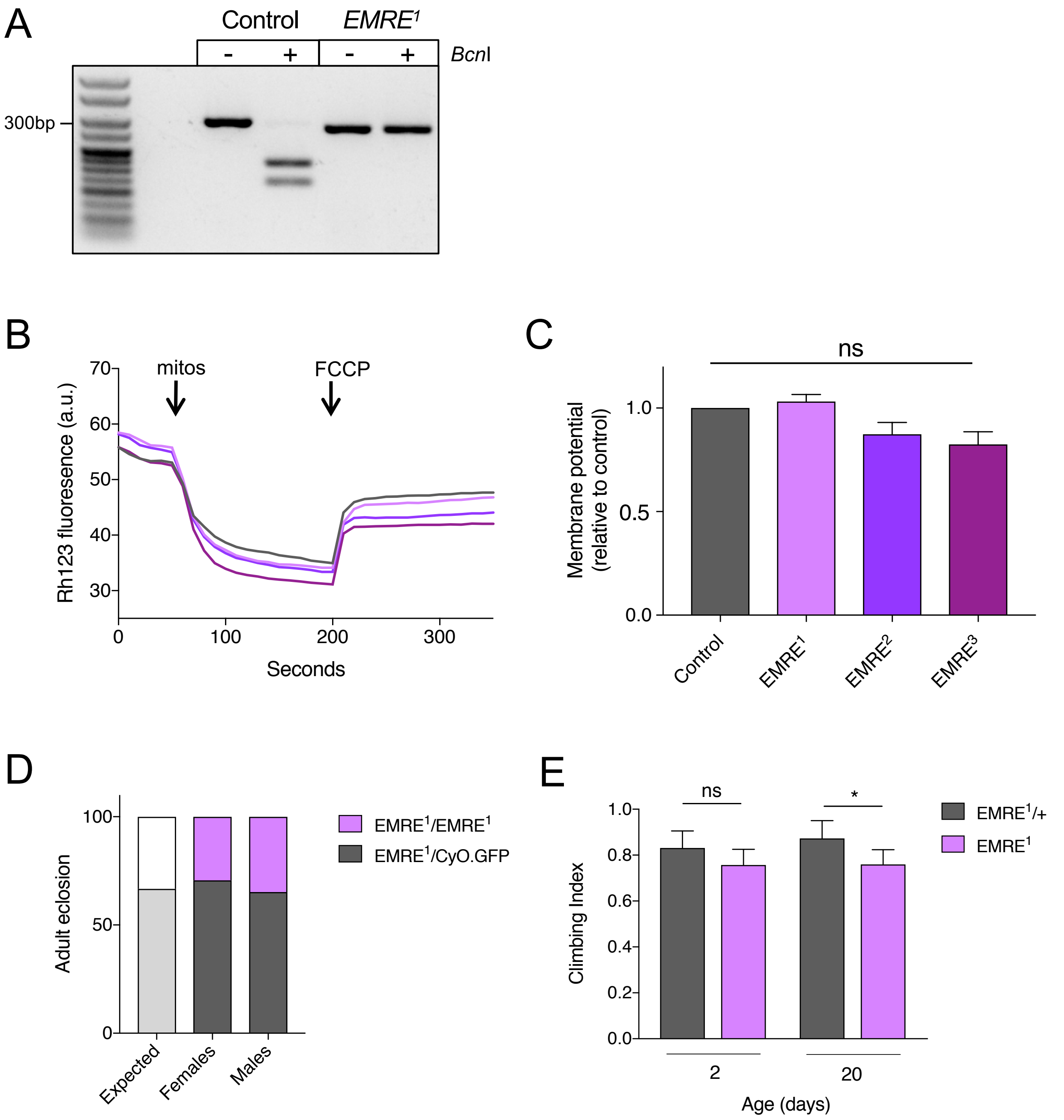

Figure S3. Additional characterisation of *EMRE*1 mutants. Related to Figure 2.
A. RFLP analysis of control and *EMRE*1 PCR products with *Bcn*I.
B. Representative traces of Rhodamine 123 (Rh123) fluorescence (a.u.: arbitrary units) in isolated mitochondria of the indicated genotypes used for the Ca^{2+} uptake assays for *EMRE* mutants.
C. Relative membrane potential was determined through Rh123 fluorescence change before and after addition of FCCP (ΔF-F0) normalized to controls (mean ± SEM; n = 3).
D. The percentage of adult flies eclosing as homozygous *EMRE*1 mutants *versus* balanced heterozygotes, together with the expected Mendelian ratio in the offspring (n > 360).
The control genotype is w^{1118}.
E. Climbing assay of *EMRE*1/+ and *EMRE*1 flies at 2 and 20 days post-eclosion. Statistical analysis: Kruskal-Wallis test with Dunn's post-hoc correction for multiple comparisons (mean ± 95% CI; n > 50; * $P < 0.05$, ns, non-significant).

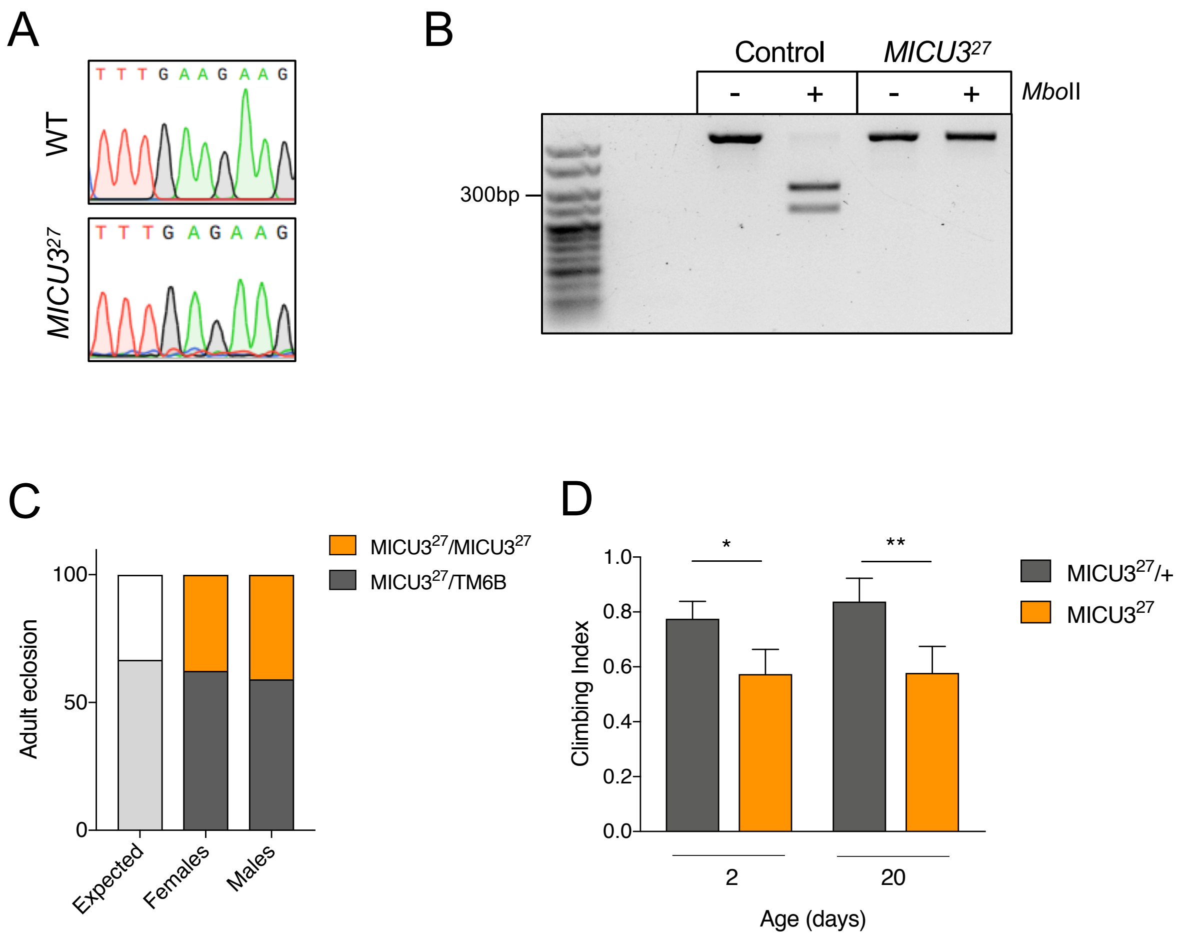

Figure S4. Additional characterisation of *MICU3*^{27} mutants. Related to Figure 4.
A. Representative sequencing chromatograms for wild-type and *MICU3*^{27} showing the single base deletion of *MICU3*^{27}.
B. RFLP analysis of control and *MICU3*^{27} PCR products with *Mbo*II.
C. The percentage of adult flies eclosing as homozygous *MICU3*^{27} mutants *versus* balanced heterozygotes, together with the expected Mendelian ratio in the offspring (n > 225).
The control genotype is w^{1118}.
D. Climbing assay of *MICU3*^{27}/+ and *MICU3*^{27} flies at 2 and 20 days post-eclosion. Statistical analysis: Kruskal-Wallis test with Dunn's post-hoc correction for multiple comparisons (mean ± 95% CI; n > 40; * $P < 0.05$, ** $P < 0.01$).

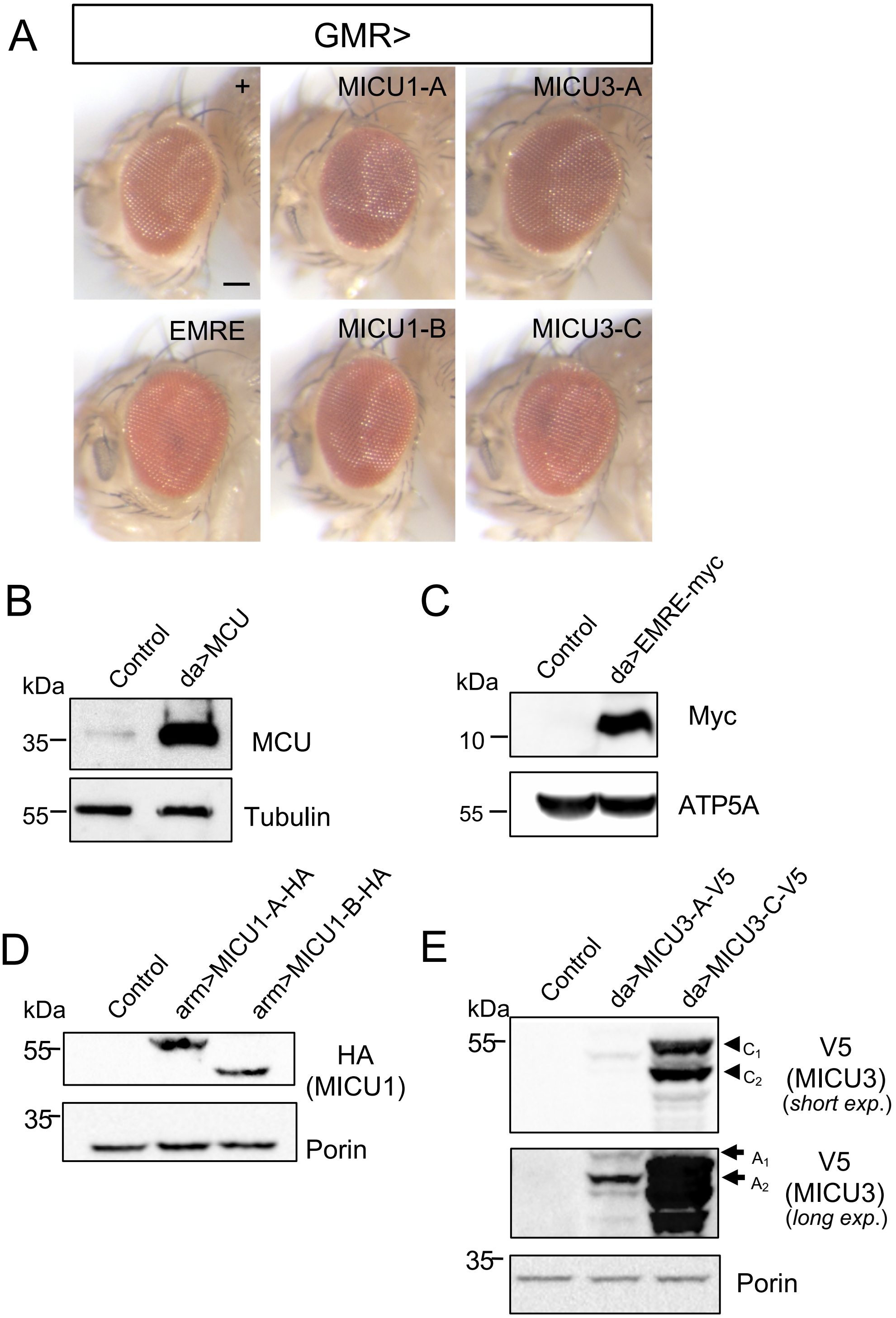

Figure S5. Analysis of transgene expression. Related to Figure 5.

A. Representative images of eye morphology for *GMR-GAL4* driven expression of individual uniporter transgenes. In all conditions the control genotype was the relevant GAL4-only genetic background control (*GAL4/+*). Scale bar = 100 µm.

B. Western blot analysis of ubiquitously (*da-GAL4*) expressed *UAS-MCU* transgene.

C. Western blot analysis of ubiquitously (*da-GAL4*) expressed *UAS-EMRE-myc* transgene.

D. Western blot analysis of transgenic MICU1 expression of *UAS-MICU1-A-3xHA* and *UAS-MICU1-C-HA* lines using a ubiquitous (*arm-GAL4*) driver.

E. Western blot analysis of relative MICU3 expression between *UAS-MICU3-A-V5* and *UAS-MICU3-C-V5* lines using a ubiquitous (*da-GAL4*) driver.

Immunoblots were probed with the indicated antibodies.

Supplementary Table 1 – qPCR Primers (linked to STAR Methods)

REAGENT or RESOURCE	SOURCE	IDENTIFIER
EMRE qPCR Forward Primer: ACATGTCCAGCGTGTACTTTC	This paper	N/A
EMRE qPCR Reverse Primer: GGTATGACGGCACAGAAGATG	This paper	N/A
javelin qPCR Forward Primer: GCGGATTTTTCCGTGAATC	*This paper*	*N/A*
javelin qPCR Reverse Primer: TCTGGCTCTGGGTGTCATC	This paper	N/A
MICU1 qPCR Forward Primer: GTGGCCATGGTCAATCTTTC	This paper	N/A
MICU1 qPCR Reverse Primer: TTGTTGCTGAGTTGGTTGTCA	This paper	N/A
MICU3 qPCR Forward Primer: GATCCACAAACCAAGCGAAT	This paper	N/A
MICU3 qPCR Reverse Primer: CCTCTTCCGGCTCTTGCT	This paper	N/A
RpL32 qPCR Forward Primer: GCCGCTTCAAGGGACAGTATCTG	This paper	N/A
RpL32 qPCR Reverse Primer: AAACGCGGTTCTGCATGAG	This paper	N/A
sulfateless qPCR Forward Primer: AAGCTGTCGATTTGAGTAGCAA	This paper	N/A
sulfateless qPCR Reverse Primer: GACTGTCCACTCGCAATCAG	This paper	N/A
