# Supplementary material for: Comprehensive Genetic Characterization of Mitochondrial Ca2+ Uniporter Components Reveals Their Different Physiological Requirements In Vivo
Source: Cell Rep. 2019 Apr 30;27(5):1541–1550.e5. doi: 10.1016/j.celrep.2019.04.033 (PMC6506686; doi:10.1016/j.celrep.2019.04.033)
Supplement: Document S2. Article plus Supplemental Information [file mmc2.pdf]

Cell Reports

Article

Comprehensive Genetic Characterization of Mitochondrial Ca^{2+} Uniporter Components Reveals Their Different Physiological Requirements *In Vivo*

Graphical Abstract

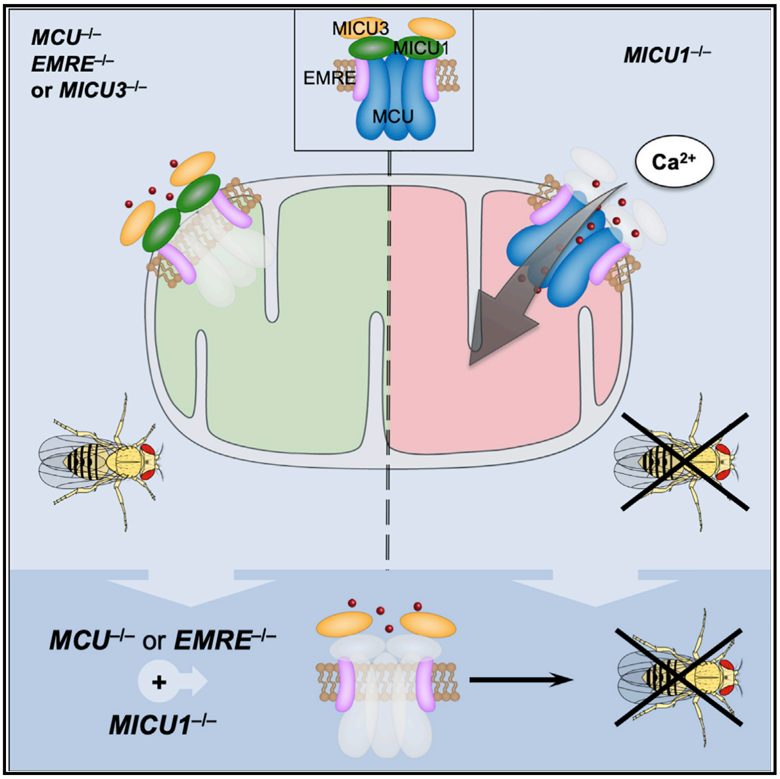

Highlights

- MCU or EMRE loss blocks fast mitochondrial calcium uptake but are relatively benign
- MCU knockout flies are short lived compared to EMRE or MICU3 mutants
- MICU1 mutants are developmentally lethal, and MCU or EMRE knockout fails to rescue
- MICU1 and MICU3 are not functionally interchangeable

Authors

Roberta Tufi, Thomas P. Gleeson, Sophia von Stockum, ..., Alvaro Sanchez-Martinez, Elena Ziviani, Alexander J. Whitworth

Correspondence

a.whitworth@mrc-mbu.cam.ac.uk

In Brief

Tufi et al. generate a genetic toolkit for all conserved components of the *Drosophila* mitochondrial calcium uniporter. Under basal conditions, *MCU* and *EMRE* loss is tolerated and *MICU3* mutants are mildly impaired. *MICU1* mutants are lethal, but this is not suppressed by *MCU* or *EMRE* loss, suggesting an unidentified uniporter-independent role.

Tufi et al., 2019, Cell Reports *27*, 1541–1550
April 30, 2019 © 2019 The Author(s).
https://doi.org/10.1016/j.celrep.2019.04.033

CellPress

OPEN ACCESS
CellPress

Comprehensive Genetic Characterization of Mitochondrial Ca^{2+} Uniporter Components Reveals Their Different Physiological Requirements *In Vivo*

Roberta Tufi,[1,4] Thomas P. Gleeson,[1,4] Sophia von Stockum,[2,3] Victoria L. Hewitt,[1] Juliette J. Lee,[1] Ana Terriente-Felix,[1] Alvaro Sanchez-Martinez,[1] Elena Ziviani,[2,3] and Alexander J. Whitworth[1,5,*]
[1]MRC Mitochondrial Biology Unit, University of Cambridge, Cambridge Biomedical Campus, Hills Road, Cambridge CB2 0XY, UK
[2]Department of Biology, University of Padova, Padova, Italy
[3]Fondazione Ospedale San Camillo, IRCCS, Lido di Venezia, Venezia, Italy
[4]These authors contributed equally
[5]Lead Contact
*Correspondence: a.whitworth@mrc-mbu.cam.ac.uk
https://doi.org/10.1016/j.celrep.2019.04.033

SUMMARY

Mitochondrial Ca^{2+} uptake is an important mediator of metabolism and cell death. Identification of components of the highly conserved mitochondrial Ca^{2+} uniporter has opened it up to genetic analysis in model organisms. Here, we report a comprehensive genetic characterization of all known uniporter components conserved in *Drosophila*. While loss of pore-forming *MCU* or *EMRE* abolishes fast mitochondrial Ca^{2+} uptake, this results in only mild phenotypes when young, despite shortened lifespans. In contrast, loss of the *MICU1* gatekeeper is developmentally lethal, consistent with unregulated Ca^{2+} uptake. Mutants for the neuronally restricted regulator *MICU3* are viable with mild neurological impairment. Genetic interaction analyses reveal that *MICU1* and *MICU3* are not functionally interchangeable. More surprisingly, loss of *MCU* or *EMRE* does not suppress *MICU1* mutant lethality, suggesting that this results from uniporter-independent functions. Our data reveal the interplay among components of the mitochondrial Ca^{2+} uniporter and shed light on their physiological requirements *in vivo*.

INTRODUCTION

The uptake of Ca^{2+} into mitochondria has long been established as a key regulator of an array of cellular homeostatic processes as diverse as bioenergetics and cell death (Granatiero et al., 2017; Mammucari et al., 2016). A series of seminal discoveries has elucidated the identity of the components that make up the mitochondrial Ca^{2+} uniporter complex. The mammalian uniporter is composed of MCU (mitochondrial calcium uniporter) as the main pore-forming protein (De Stefani et al., 2011; Baughman et al., 2011); its paralog MCUb (Raffaello et al., 2013); a small structural component, EMRE (essential MCU regulator) (Sancak et al., 2013); and the regulatory subunits MICU1–MICU3 (mitochondrial calcium uptake 1–3) (Perocchi et al., 2010; Plovanich et al., 2013; Patron et al., 2019). Reconstitution studies in yeast, which lacks a mitochondrial Ca^{2+} uniporter, have demonstrated that heterologous co-expression of MCU and EMRE is necessary and sufficient to confer uniporter activity (Kovács-Bogdán et al., 2014). The family of EF-hand-containing proteins (MICU1, MICU2, and MICU3) has been shown to exhibit a gatekeeper function for the uniporter, inhibiting Ca^{2+} uptake at low cytoplasmic concentrations (Mallilankaraman et al., 2012; Kamer et al., 2017; Patron et al., 2019). These components are generally highly conserved across eukaryotes, including most metazoans and plants, but not in many fungi and protozoans, reflecting their ancient and fundamental role (Bick et al., 2012).

Although the composition and function of the uniporter have been well characterized *in vitro* and in cell culture models, the physiological role of the uniporter is beginning to emerge with *in vivo* characterization of knockout mutants (Liu et al., 2017). Current data present a complex picture. Initial studies of *MCU* knockout mice described a viable strain with a modest phenotype in a mixed genetic background (Pan et al., 2013), although subsequent studies using an inbred background reported *MCU* loss to be lethal or semi-viable (Murphy et al., 2014) and tissue-specific conditional knockout revealed an important role in cardiac homeostasis (Luongo et al., 2015). Similarly, loss of *MICU1* in mice has a complex phenotype, varying from fully penetrant perinatal lethality (Antony et al., 2016) to incomplete lethality with a range of neuromuscular defects that unexpectedly improve over time in surviving animals (Liu et al., 2016).

One explanation for the reported phenotypic variability is that perturbing mitochondrial Ca^{2+} uptake can be influenced by additional factors, the most obvious being genetic background. Hence, there is a need for greater investigation into the physiological consequences of genetic manipulation of the uniporter components in a genetically powerful model system. Here we report a comprehensive genetic analysis of the uniporter complex components that are conserved in *Drosophila*. This includes loss-of-function mutants for *MCU*, *EMRE*, *MICU1*, and *MICU3* (*Drosophila* lack *MCUb* and *MICU2*) and corresponding inducible transgenic expression lines. Despite lacking fast Ca^{2+} uptake, *MCU* and *EMRE* mutants present a surprising lack of

This is an open access article under the CC BY license (http://creativecommons.org/licenses/by/4.0/).

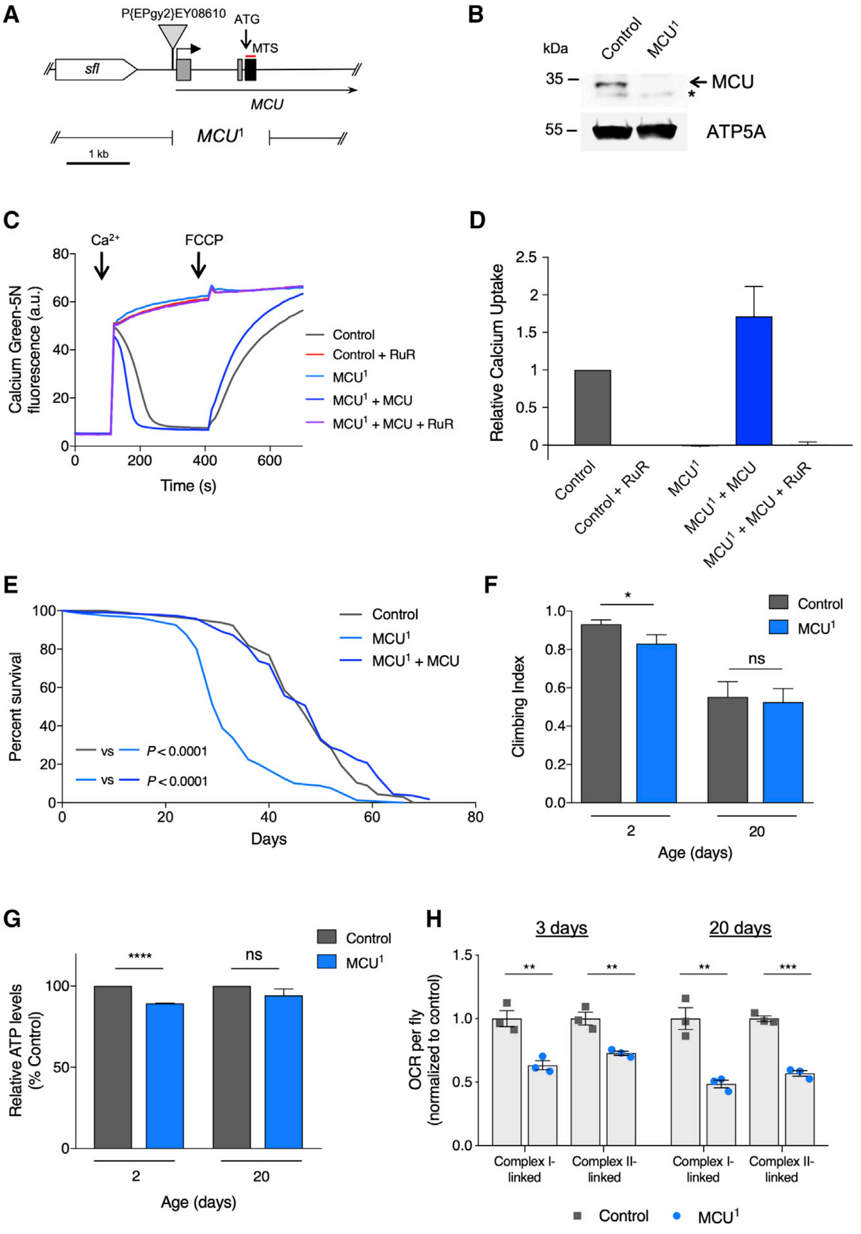

Figure 1. The MCU^1 Mutant Abolishes Fast Mitochondrial Ca^{2+} Uptake and Shortens Lifespan without Affecting Organismal Phenotypes despite Respiratory Defects

(A) Overview of the *MCU* (CG18769) 5′ gene region (from FlyBase), including the neighboring *sfl* (*sulfateless*). The P{EPgy2}EY08610 transposable element used to generate MCU^1 is displayed, along with the location of the MCU^1 breakpoints.

(B) Western blot analysis of MCU^1. Immunoblots were probed with the indicated antibodies. The asterisk denotes a non-specific band. Mitochondrial ATP5A is used as a loading control.

(C) Representative traces of Ca^{2+} uptake in mitochondria isolated from adult flies of the indicated genotypes after addition of 45 μM $CaCl_2$. Extra-mitochondrial Ca^{2+} was measured by calcium green-5N fluorescence. Ca^{2+} was released from mitochondria by addition of 1 μM FCCP. Addition of the MCU inhibitor ruthenium red (RuR; 2 μM) blocks mitochondrial Ca^{2+} uptake, which is mirrored by MCU^1. Mitochondrial Ca^{2+} uptake is restored by transgenic re-expression of *MCU* driven by *da-GAL4*.

(D) Relative uptake kinetics of traces shown in (C) were determined through linear fits of the initial phase of Ca^{2+} uptake and normalized to the wild-type control (mean ± SEM, n = 3).

(E) Lifespan curves of MCU^1 male flies compared with control and transgenic rescue (MCU^1 + *MCU*) driven by *da-GAL4*. Statistical analysis: Mantel-Cox log-rank test (n ≥ 80).

(F) Climbing assay of control (a precise excision revertant, MCU^{rv}) and MCU^1 flies 2 and 20 days post-eclosion. Significance measured by Kruskal-Wallis test with Dunn's post hoc correction for multiple comparisons (mean ± 95% confidence interval (CI); n > 70; *p < 0.05; ns, non-significant).

(G) Relative ATP levels from control and MCU^1 flies. Statistical analysis: unpaired t test (mean ± SD; n = 2–3; ****p < 0.0001; ns, non-significant).

(H) Oxygen consumption rate (OCR) of control and MCU^1 flies at 3 and 20 days post-eclosion. Statistical analysis: unpaired t test (mean ± SEM; n = 3; **p < 0.01, ***p < 0.001).

The control genotype is w^{1118} unless otherwise stated. See also Figures S1 and S2.

organismal phenotypes, although both mutants are short lived, with a more pronounced effect when MCU is lost. In contrast, loss of *MICU1* causes developmental lethality, whereas mutants for *MICU3* are viable with modest phenotypes. Performing genetic interaction studies with these strains, we confirm the gatekeeper function of MICU1 is conserved in flies and reveal that *MICU1* and *MICU3* are not functionally interchangeable. More surprisingly, we find that loss of *MCU* or *EMRE* does not suppress *MICU1* mutant lethality, suggesting that the lethality results from MCU-independent functions. The generation of these genetic tools in *Drosophila* will facilitate further investigation of the functional roles of the uniporter components *in vivo*.

RESULTS

To generate null mutants for *MCU*, we used a P element mobilization technique exploiting a transposon at the 5′ end, P{EPgy2}$MCU^{EY08610}$ (Figure 1A). We isolated a single imprecise excision: a deletion of 1,557 bp removing the 5′ end of *MCU* that includes the first three exons containing the start codon and mitochondrial targeting sequence common to most isoforms. We refer to this mutation as MCU^1 (Figure 1A). Precise excision revertants were also recovered (see STAR Methods). The MCU^1 deletion can be detected by genomic PCR, and the breakpoints were verified by Sanger sequencing (Figure S1A). The neighboring genes, *sulfateless* (*sfl*) and *javelin* (*jv*), remained intact

and showed unaltered levels of expression (Figures S1B and S1C). Immunoblot analysis of crude mitochondrial extracts from homozygous *MCU*[1] mutant homogenates using an antibody raised against the C terminus of *Drosophila* MCU confirmed the absence of MCU protein (Figure 1B).

Mitochondria from human or mouse cells lacking MCU fail to perform fast Ca^{2+} uptake (Baughman et al., 2011; De Stefani et al., 2011; Pan et al., 2013). To verify that *MCU*[1] represents a functional null mutant, mitochondria were isolated from homozygous *MCU*[1] adult flies and assayed for Ca^{2+} uptake. Similar to mammalian cells, the addition of Ca^{2+} to purified, energized mitochondria from wild-type *Drosophila* yields a rapid spike of extra-mitochondrial calcium green-5N fluorescence followed by a progressive decline in fluorescence as Ca^{2+} is buffered by mitochondria (Figures 1C and 1D). Ca^{2+} is released again upon depolarization by the uncoupling agent carbonyl cyanide-4-(trifluoromethoxy)phenylhydrazone (FCCP), as reflected by the concomitant rise in calcium green-5N fluorescence. As expected, rapid Ca^{2+} uptake is blocked by the addition of the MCU inhibitor ruthenium red (RuR). This effect is fully replicated in *MCU*[1] mutant mitochondria, reflecting a complete loss of fast Ca^{2+} uptake. The lack of Ca^{2+} uptake was not due to loss of membrane potential, because this was equivalent across the samples (Figures S1D and S1E). Moreover, Ca^{2+} uptake is restored upon transgenic expression of *MCU* (Figures 1C and 1D; Figures S1D and S1E). Altogether, these data show that *MCU*[1] is a null mutant incapable of fast Ca^{2+} uptake.

Despite this deficiency, *MCU*[1] mutants are homozygously viable and develop to adult stage in expected Mendelian proportions (Figure S1F). However, *MCU*[1] mutants are significantly shorter lived than controls (~34% reduction of median lifespan), a phenotype that is fully rescued by ubiquitous expression of transgenic *MCU* (Figure 1E). Despite this attenuated longevity, *MCU*[1] mutants do not display an appreciable decline in vitality, as assessed by analyzing their motor ability using a negative geotaxis (climbing) assay (Figure 1F).

We next sought to determine the effect of MCU loss on mitochondrial metabolic function. Young *MCU*[1] mutants showed a modest but significant decrease in basal ATP levels compared to controls (Figure 1G), which ameliorated with age. In contrast, measurement of oxygen consumption rate (OCR) revealed a marked reduction in complex I- or complex II-linked respiration in young and older flies (Figure 1H). Assessing the impact on mitochondrial cell biology, we found no difference in mitochondrial morphology in flight muscle (Figure S2A) or mitochondrial axonal transport (Figures S2B and S2C). Altogether, these results indicate that loss of MCU affects mitochondrial respiratory capacity, but this is surprisingly well tolerated at the organismal level.

To mutate *EMRE*, we used a CRISPR/Cas9-based approach (Port et al., 2014) with two simultaneously expressed transgenic guide RNAs (gRNAs). We isolated several insertion/deletion (indel) events resulting in frameshift mutations that led to premature stop codons. Three such mutations are shown in Figure 2A. As with *MCU*[1], analysis of these *EMRE* mutants revealed that they all exhibit no fast mitochondrial Ca^{2+} uptake (Figures 2C and 2D), in normally energized mitochondria (Figures S3B and S3C), indicating that the three mutants are functionally equivalent. We focused on one mutant, *EMRE*[1], whose mutation abolishes a *Bcn*I restriction site (Figure S3A) and shows a substantial reduction in the level of the mRNA transcript (Figure 2B), for further characterization.

Similar to the *MCU*[1] flies, *EMRE*[1] mutants are viable, eclose at expected Mendelian ratios (Figure S3D), and display a significantly shortened lifespan (23% reduction of median lifespan compared to control) (Figure 2E). The climbing ability of *EMRE*[1] mutants was similar to that of heterozygous controls at 2 days old, although a modest difference becomes apparent by 20 days (Figure S3E). This is mirrored in the basal ATP level of *EMRE*[1] mutants, being only marginally reduced at 20 days (Figure 2F). However, in contrast to the strong reduction in respiration seen in *MCU*[1] mutants, the complex I- or complex II-linked respiration of *EMRE*[1] flies is either non-significant or only modestly affected compared to controls (Figure 2G).

To target *MICU1*, we again used P element mobilization, using P{SUPor-P}*MICU1*^{KG04119}, and isolated a large deletion spanning ~11 kb, removing half of *MICU1* and extending some 9 kb upstream of *MICU1* (Figure 3A). This region is relatively gene sparse and devoid of additional predicted protein-coding genes. Expression analysis of homozygous *MICU1*[32] larvae yielded no detectable transcript, establishing it as a null allele (Figure 3B). In contrast to *MCU* and *EMRE* mutants, homozygous *MICU1*[32] mutants are larval lethal, with a few animals reaching the third instar stage. Supporting this, ubiquitous expression of two independent RNAi transgenes also caused developmental lethality.

Because *MICU1*[32] mutants do not reach adulthood, we analyzed larval locomotion (crawling) as a measure of organismal vitality, revealing a behavioral deficit (Figure 3C). We further found that these mutants had significantly lower ATP levels compared to controls (Figure 3D), indicative of a substantial mitochondrial impairment. This prompted us to investigate other indicators of mitochondrial homeostasis. Visualizing mitochondria in larval epidermal cells, the morphology looked comparable to controls, although the mitochondria were more diffuse in the *MICU1*[32] mutants occupying a smaller area of the cell (Figures 3E and 3F). Furthermore, mitochondrial axonal transport was significantly reduced in *MICU1*[32] larvae (Figures 3G and 3H). Collectively, these data reveal the presence of multiple mitochondrial defects that together might be responsible for lethality of the *MICU1*[32] mutants.

MICU1[32] lethality was fully rescued upon ubiquitous expression of hemagglutinin (HA)-tagged MICU1 by either the A or the B isoform (Figure 3I). In addition, organismal vitality, as measured by climbing ability, was restored (Figure 3J), demonstrating that these phenotypes are specifically due to loss of *MICU1*.

Drosophila do not have an ortholog of the *MICU1* paralog *MICU2*, but *MICU3* is conserved, encoded by *CG4662* (Figure 4A). Similar to mammals (Patron et al., 2019), *Drosophila* *MICU3* also appears to be mainly expressed in neuronal tissue (see FlyBase and Graveley et al., 2011). Little is known about the function of MICU3, and no *in vivo* studies have been reported. To assess its role *in vivo*, we used CRISPR/Cas9 to induce indel mutations. One of these mutations, *MICU3*[27], a single-base deletion (Figure 4B; Figure S4A) that abolishes a MboII restriction site (Figure S4B), leads to a frameshift and early truncation. This mutation also substantially de-stabilizes the *MICU3*

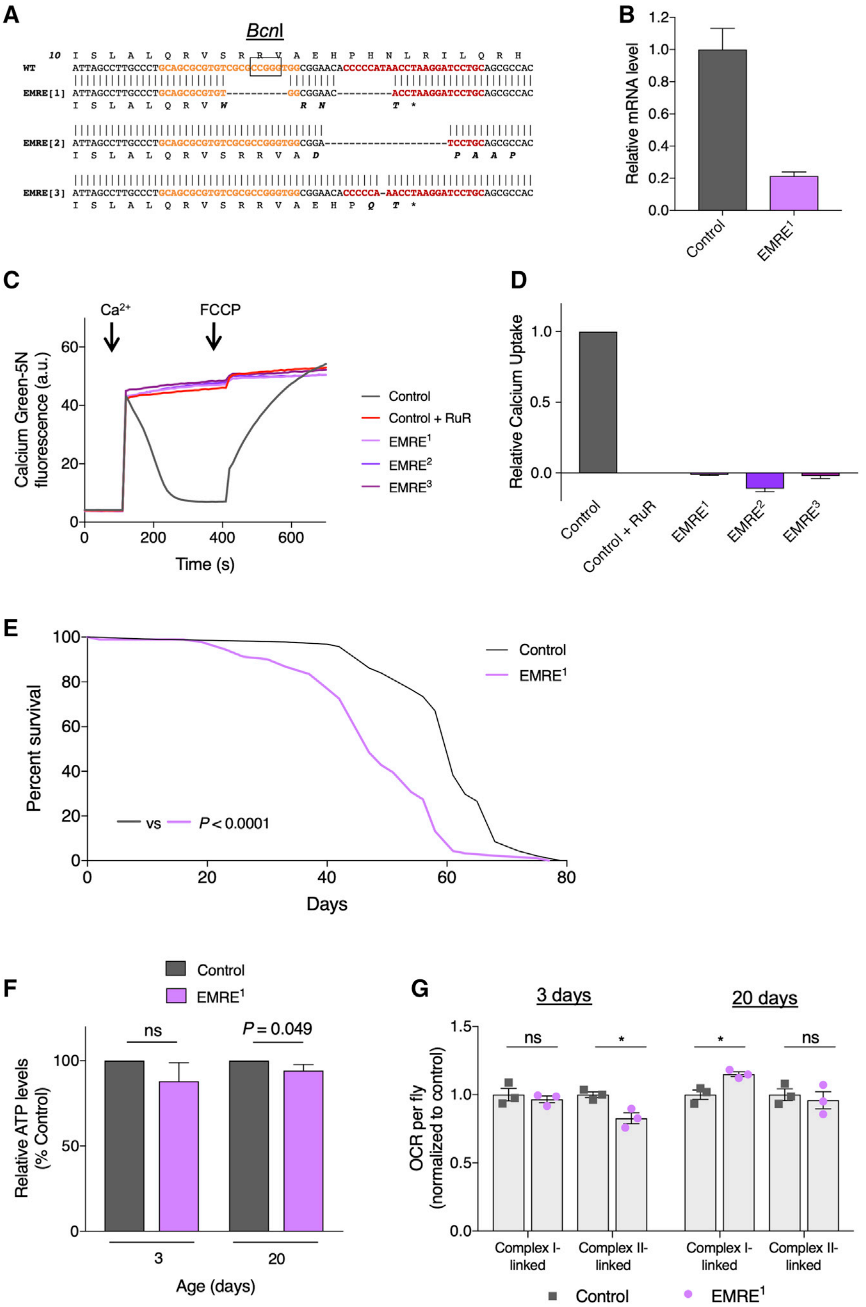

Figure 2. *EMRE* Mutants Exhibit No Fast Mitochondrial Ca^{2+} Uptake and Are Short Lived but Have Mild Phenotypes

(A) Sequence alignments of wild-type and *EMRE* mutants, with predicted protein sequences and positions of gRNA recognition sites (colored text). the box denotes the *Bcn*I cleavage site.

(B) Relative expression of *EMRE* transcript for control and $EMRE^{1}$ mutants (mean ± SD; n = 3).

(C) Representative traces of Ca^{2+} uptake in mitochondria isolated from adult flies of the indicated genotypes after addition of 45 μM $CaCl_2$. Extra-mitochondrial Ca^{2+} was measured by calcium green-5N fluorescence. Ca^{2+} was released from mitochondria by addition of 1 μM FCCP. *EMRE* mutations prevent mitochondrial Ca^{2+} uptake equivalent to the inhibitor ruthenium red (RuR; 2 μM).

(D) Relative uptake kinetics were determined through linear fits of Ca^{2+} uptake traces and normalized to controls (mean ± SEM; n = 3).

(E) Lifespan curves of $EMRE^{1}$ male flies compared with control. Statistical analysis: Mantel-Cox log-rank test (n ≥ 91).

(F) Relative ATP levels from control and $EMRE^{1}$ flies. Statistical analysis: unpaired t test (mean ± SD; n = 3; ns, non-significant).

(G) Oxygen consumption rate (OCR) of control and $EMRE^{1}$ flies at 3 and 20 days post-eclosion. Statistical analysis: unpaired t test (mean ± SEM; n = 3; *p < 0.05).

The control genotype is w^{1118} in all cases. See also Figure S3.

transcript (Figure 4C). In contrast to loss of *MICU1*, homozygous $MICU3^{27}$ mutants are fully viable (Figure S4C), though lifespan was modestly (7% reduction in median lifespan) but significantly reduced (Figure 4D). In addition, these mutants exhibited a significant climbing defect in young and older flies (Figure S4D). These results indicate a function for MICU3 in proper maintenance of neuronal function, consistent with its neuronally restricted expression. However, analyzing mitochondrial respiration from heads of $MICU3^{27}$ mutants, we observed no significant differences in young and older flies compared to control (Figure 4E).

To investigate the functional relationships among the various uniporter components, we undertook several genetic interaction studies. First, because *Drosophila* MICU1 and MICU3 share a fair degree of homology (~49% similarity and ~31% identity between MICU1-B and MICU3-C), we reasoned that they may share some functional overlap. To address this, we asked whether normally neuronally restricted MICU3 could functionally substitute for MICU1. Thus, we ectopically expressed *MICU3* ubiquitously in $MICU1^{32}$ mutants. Here we chose to express isoforms A and C, because these cover all predicted coding regions (Figure 4A). However, neither MICU3 isoform was able to restore viability of $MICU1^{32}$ mutants or shift the lethal phase (Figure 3I), indicating that MICU3 is not functionally equivalent to MICU1 *in vivo*.

MICU1 has been shown to provide a gatekeeper function for the uniporter channel, with loss of MICU1 causing unregulated mitochondrial Ca^{2+} uptake. It has also been shown that in mice lacking *MICU1*, genetic reduction of *EMRE* substantially ameliorates the *MICU1* phenotypes (Liu et al., 2016). Thus, we reasoned that the lethality of the $MICU1^{32}$ mutants is caused by unregulated Ca^{2+} entry, which should be prevented by loss

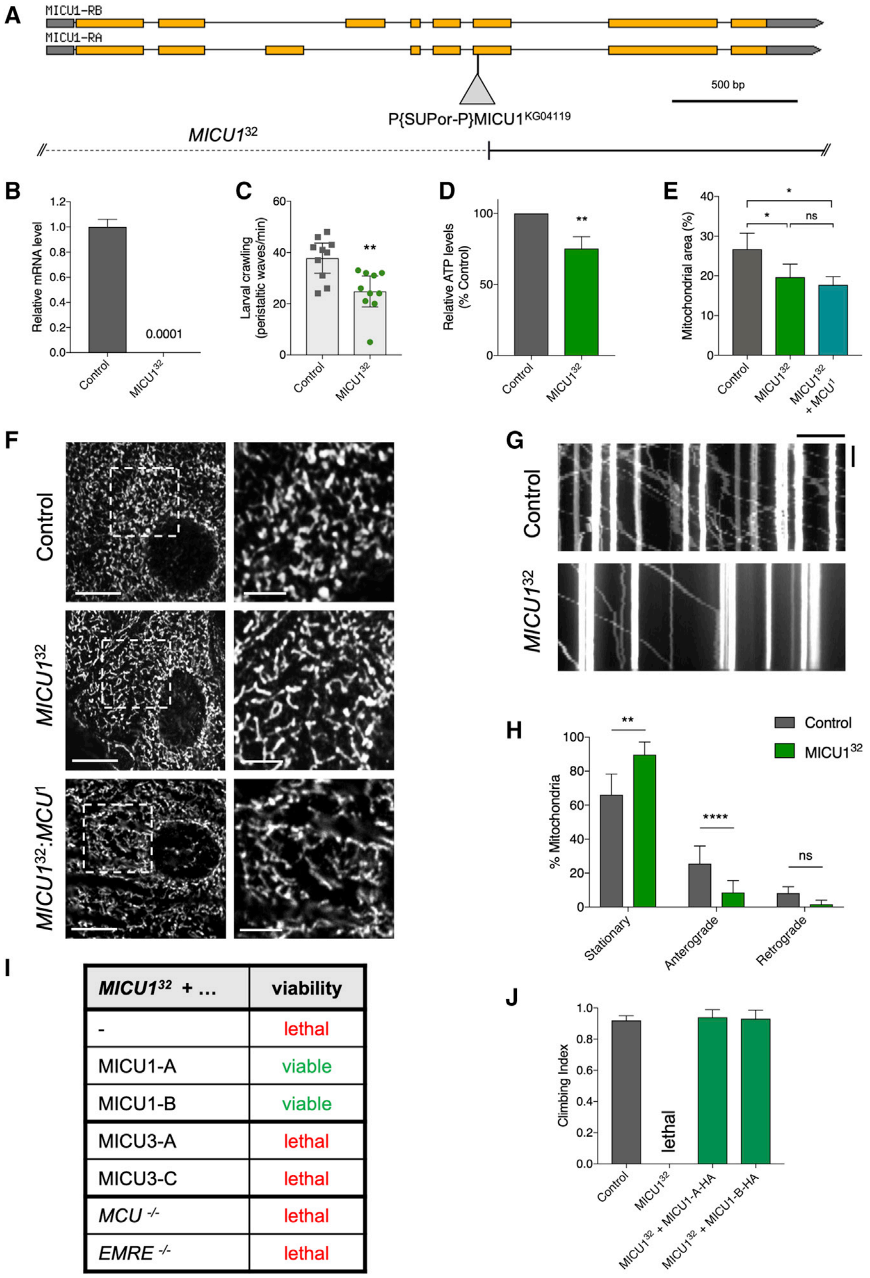

*MICU1*³² + ...	viability
-	lethal
MICU1-A	viable
MICU1-B	viable
MICU3-A	lethal
MICU3-C	lethal
MCU -/-	lethal
EMRE -/-	lethal

Figure 3. *MICU1*³² Mutants Are Lethal, Have Reduced ATP and Mitochondrial Transport, and Are Not Rescued by *MCU*¹ or *EMRE*¹

(A) Overview of the *MICU1* (CG4495) gene region (from FlyBase). The P{SUPor-P}MICU1^{KG04119} transposable element used to generate *MICU1*^{32} is displayed, as well as the deleted region in *MICU1*^{32}.

(B) Relative expression of *MICU1* transcript for control and *MICU1*^{32} larvae (mean ± SD; n = 3).

(C) Larval crawling of control and *MICU1*^{32} larvae, expressed as the number of peristaltic waves per minute. Statistical analysis: unpaired t test (mean ± 95% CI; n = 10; **p = 0.0034).

(D) Relative ATP levels from control and *MICU1*^{32} larvae. Statistical analysis: unpaired t test (mean ± SD; n = 4; **p = 0.0011).

(E) Quantification of mitochondrial density in the images shown in (F) (zoomed images). Statistical analysis: one-way ANOVA (mean ± SD; n = 4; *p < 0.05; ns, non-significant).

(F) Confocal microscopy analysis of epidermal cells in control and *MICU1*^{32} larvae immunostained with the mitochondrial marker anti-ATP5A. Boxed areas are enlarged to the right. Scale bars: 10 μm (left), 4 μm (right).

(G) Representative kymographs of mitochondrial axonal transport in control and *MICU1*^{32} larvae. Scale bars: 10 μm (horizontal), 50 s (vertical). Genotypes—control: *M12-GAL4, UAS-mito-HA-GFP/+*. *MICU1*^{32}*: MICU1*^{32}*/MICU1*^{32}*; M12-GAL4, UAS-mito-HA-GFP/+*.

(H) Quantification of mitochondrial transport shown in (G). Statistical analysis: one-way ANOVA (mean ± 95% CI; n = 6 (control) and 11 (mutant); **p < 0.01, ****p < 0.0001; ns, non-significant).

(I) Table of viability of *MICU1*^{32} rescue by transgenic expression of *MICU1* or *MICU3* isoforms or loss of *MCU* or *EMRE*. Transgenic expression was induced using ubiquitous drivers: *arm-GAL4* for *MICU1* and *da-GAL4* for *MICU3*.

(J) Climbing assay of control flies (*arm-GAL4*/+) and *MICU1*^{32} mutants with ubiquitous (*arm-GAL4*) driven transgenic re-expression of HA-tagged MICU1-A and MICU1-B isoforms. Statistical analysis: Kruskal-Wallis test with Dunn's post hoc correction for multiple comparisons (mean ± 95% CI; n ≥ 40).

The control genotype is w^{1118} unless otherwise stated.

of the MCU channel. To test this, we combined homozygous *MICU1*^{32} and *MCU*^{1} mutants and, to our surprise, found that this did not suppress the lethality or noticeably shift the lethal phase (Figure 3I). We corroborated this finding by combining *MICU1*^{32} mutants with *EMRE*^{1} mutants, with the same result (Figure 3I). Moreover, the aberrant mitochondrial distribution in *MICU1*^{32} epidermal cells was not restored by loss of MCU (Figures 3E and 3F).

Overexpression paradigms disrupting uniporter stoichiometry have previously been used to study the functional relationships of uniporter components (Choi et al., 2017). Using a classic eye morphology assay as a readout of the impact of genetic interactions on cell and tissue viability, we first found that overexpression of any of the uniporter components alone in the eye, using a *GMR-GAL4* driver, had no effect on eye or ommatidial morphology (Figure S5A), with overexpression confirmed by immunoblotting (Figures S5B–S5E). This indicates that overabundance of any uniporter component, including MCU, is insufficient to grossly disrupt mitochondrial Ca^{2+} homeostasis as expected. However, the co-expression of *MCU* and *EMRE* caused a dramatic disruption of eye morphology with a general loss of retinal pigment and ommatidia, resulting in a glazed

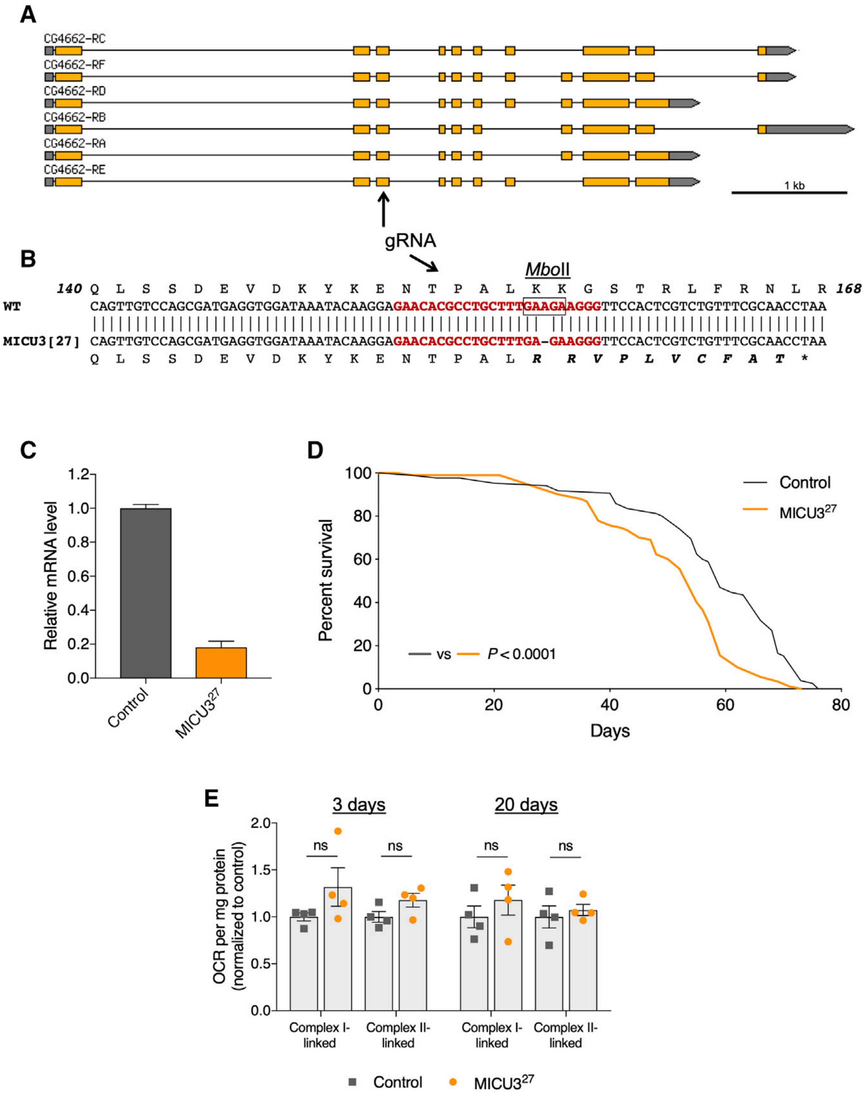

Figure 4. *MICU3* Loss of Function Is Largely Benign

(A) Overview of *MICU3* (CG4662) gene region (from FlyBase), including positions of the gRNA recognition site.

(B) Sequence alignment of wild-type and *MICU3*^{27}, with relative predicted protein sequences. The gRNA recognition site is highlighted in red. The box denotes the MboII cleavage site.

(C) Relative expression of *MICU3* transcript for control and *MICU3*^{27} flies (mean ± SD; n = 3).

(D) Lifespan curves of *MICU3*^{27} male flies compared with control. Statistical analysis: Mantel-Cox log-rank test (n ≥ 84).

(E) Oxygen consumption rate (OCR) of control and *MICU3*^{27} flies at 3 and 20 days post-eclosion. Statistical analysis: unpaired t test (mean ± SEM; n = 4).

The control genotype is w^{1118} in all cases. See also Figure S4.

appearance with occasional black, necrotic patches (Figure 5A). This effect is consistent with the cooperative actions of MCU and EMRE to create the channel, and in line with reconstitution experiments in yeast showing that expression of mammalian *MCU* and *EMRE* are necessary and sufficient to elicit Ca^{2+} uniporter activity. The gross disruption of eye integrity also demonstrates the catastrophic effects of unregulated mitochondrial Ca^{2+} entry.

In contrast, co-expression of *MCU* with either *MICU1* or *MICU3* was not so detrimental, although in all cases, it caused a mild disruption of the ommatidial arrangement, resulting in a mild roughened appearance (Figure 5A). This system allowed us to test possible functional differences among the isoforms of *MICU1* and *MICU3*. In general, the co-expression of all isoforms with *MCU* caused similar effects, though the expression of *MICU3-C* gave a slightly stronger phenotype that may reflect its greater level of expression compared to *MICU3-A* (Figure S5E). The enhanced phenotype of *MCU:MICU3* co-expression is consistent with a report that MICU3 enhances MCU-mediated Ca^{2+} uptake (Patron et al., 2019).

We next reasoned that if the dramatic eye phenotype caused by *MCU* and *EMRE* co-expression was due to extra unregulated channels and excessive Ca^{2+} uptake, this may be ameliorated by co-expression of the *MICU1* gatekeeper. Co-expression of either *MICU1-A* or *MICU1-B* with *MCU* and *EMRE* prevented the *MCU:EMRE* phenotype (Figure 5B). Co-expression of a *GFP* control confirmed that this was not due to titration of multiple upstream activating sequence (UAS) lines. Although both isoforms provided excellent rescue, isoform B appeared slightly more effective than isoform A. These observations are consistent with MICU1 providing the main gatekeeper function for the uniporter.

We also used this assay to test the functionality of MICU3 in this context. In contrast to *MICU1* expression, co-expression of either *MICU3-A* or *MICU3-C* with *MCU* and *EMRE* provided no suppressing effect for the *MCU:EMRE* phenotype (Figure 5B). These results are consistent with MICU3 not being a major uniporter gatekeeper *in vivo*.

DISCUSSION

The capacity of mitochondria to take up Ca^{2+} has important implications for cellular homeostasis as it regulates fundamental processes from metabolism to cell death. In this context, the mitochondrial Ca^{2+} uniporter plays a crucial function, driving the rapid entry of Ca^{2+} into mitochondria. To better understand the physiological role of mitochondrial Ca^{2+} uptake, we have used the genetically powerful model *Drosophila* to manipulate

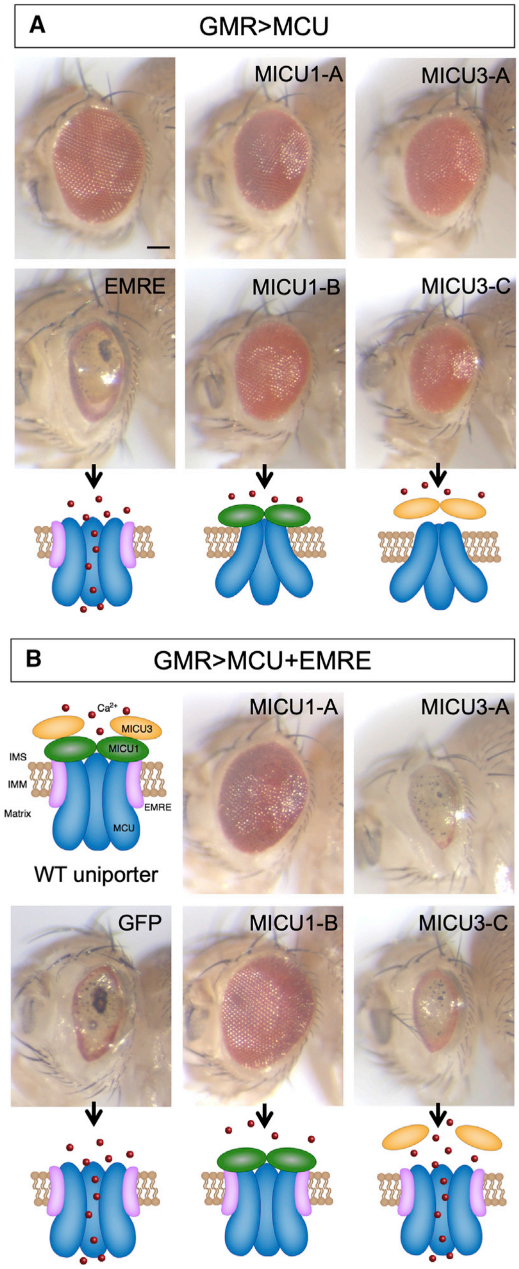

Figure 5. Genetic Interactions of Overexpression Uniporter Components

(A) Flies bearing the eye-specific *GMR-GAL4* driver and *UAS-MCU* transgene (GMR > MCU) crossed to transgenes of the indicated uniporter components or to a control (+) w^{1118} line.

(B) Flies bearing a combination of the eye-specific *GMR-GAL4* driver and both *UAS-MCU* and *UAS-EMRE* transgenes (GMR > MCU+EMRE) crossed to transgenes of the indicated components or to a control (GFP) transgene.

Below the micrograph images are schematic cartoons of the proposed status of the uniporter based on the composition of the overexpressed components. Scale bar: 100 μm. See also Figure S5.

the conserved set of dedicated channel and regulatory proteins that form the uniporter complex.

MCU mutants are viable and fertile with no gross morphological or behavioral defects, which was initially surprising given the historical importance of mitochondrial Ca^{2+}. Still, this corroborates another report of fly *MCU* mutants (Choi et al., 2017) and is consistent with studies in mice and worms in which deletion of *MCU* orthologs is essentially benign at the organismal level under basal conditions (Pan et al., 2013; Xu and Chisholm, 2014). However, fly *MCU* mutants are significantly shorter lived than controls. This situation is mirrored by *EMRE* mutants, albeit with a smaller impact on lifespan. The reason for the shortened lifespans is unknown but may reflect the effects of a chronic bioenergetic deficit evident from the OCR measurements. Accordingly, *MCU* mutants show a greater respiration defect compared to *EMRE* mutants, consistent with their respective impacts on lifespan. The respiratory impairment could be due to the previously reported increase in oxidative stress that occurs in *MCU* mutants (Choi et al., 2017), which has yet to be assessed in *EMRE* mutants. Alternatively, the short lifespan may be due to a myriad of potential metabolic imbalances, such as disruption of NADH/NAD+ levels. Chronic adaptations may also occur through transcriptional responses. Further studies analyzing the metabolic and transcriptional changes occurring in these flies will shed light on this fundamental question.

Nevertheless, the *EMRE* mutants are relatively benign at the organismal level, which corroborates the surprising viability of *MCU* mutants. Considering this, it is striking that flies, like mice and worms, consistently show an ability to compensate for the lack of fast mitochondrial Ca^{2+} uptake, suggesting the induction of some adaptive mechanism, as discussed by others (Murphy et al., 2014; Harrington and Murphy, 2015). While alternative routes of mitochondrial Ca^{2+} entry must exist, because matrix Ca^{2+} is not abolished in *MCU* knockout (KO) mice (Pan et al., 2013), proposed mechanisms are speculative, and it is unclear whether they constitute a compensatory adaptation for fast Ca^{2+} uptake or simply allow gradual, slow accumulation (Harrington and Murphy, 2015). However, rapid mitochondrial Ca^{2+} uptake mediated by MCU is thought to constitute a specific metabolic regulatory mechanism, e.g., to increase ATP production, under certain conditions, such as strenuous exercise or pathological conditions (Denton, 2009), which is partly evident in the *MCU* KO mice (Pan et al., 2013) or heart-specific conditional KO (Luongo et al., 2015). Such important physiological roles would not necessarily be apparent under basal conditions in flies. MCU has also been proposed to promote wound healing (Xu and Chisholm, 2014); however, our preliminary studies did not find evidence supporting this. The current study presents a summary of the requirements of uniporter components under basal conditions, and further work will be needed to evaluate the role of the uniporter in the full range of physiological conditions.

In seeking to understand the importance of the regulatory components of the uniporter, we also developed loss-of-function models for *MICU1* and *MICU3*. In contrast to *MCU* and *EMRE* mutants, loss of *MICU1* results in larval lethality, which is associated with alterations in mitochondrial distribution and motility, and a reduced level of total ATP. In line with its role as

the principle gatekeeper of the uniporter, coupled with excess mitochondrial Ca^{2+} triggering cell death, we reasoned that the lethality was due to Ca^{2+} accumulation in the mitochondrial matrix through unregulated MCU-EMRE channels. Supporting this, we observed that dual overexpression of *MCU* and *EMRE* in the eye leads to substantial loss of retinal tissue; concomitant overexpression of *MICU1* is sufficient to prevent this phenotype, consistent with MICU1 re-establishing appropriately regulated uniporter channels.

However, one observation that was most surprising to us was the inability of *MCU* or *EMRE* mutants to rescue the *MICU1* mutant lethality. This result is particularly puzzling, because it has been shown that mice lacking *MICU1*, which present multiple pathogenic phenotypes, are substantially rescued by genetic reduction of *EMRE* levels (Liu et al., 2016). While the reason for the lack of rescue in flies is unclear, we postulate that this suggests the function of MICU1 is not limited to uniporter-dependent Ca^{2+} uptake. We do not know whether the lethality of *MICU1* mutants is specifically due to excessive mitochondrial Ca^{2+} levels; however, it appears to be independent of fast mitochondrial Ca^{2+} uptake, because this is eliminated in *MCU* and *EMRE* mutants. As noted earlier, other routes of Ca^{2+} uptake into mitochondria exist, but the mechanisms that regulate them are uncertain. It is possible that aberrant manganese uptake, as reported to occur in cell models, may contribute to the *MICU1* mutant lethality (Kamer et al., 2018; Wettmarshausen et al., 2018). However, this mechanism would presumably be expected to be mitigated by loss of MCU. Nevertheless, these *Drosophila* models are ideally suited for unbiased genetic screening to uncover such fundamental regulatory mechanisms.

In contrast to *MICU1*, loss of *MICU3* was well tolerated overall at the organismal level. Functional analysis of MICU3 is extremely limited, but the neuronally restricted expression led us to anticipate that these mutants might have more neurological-specific phenotypes, which was at least partly borne out. Whereas longevity of these mutants was only minimally affected, they exhibited a notable locomotor deficit even in young flies. We initially hypothesized that MICU3 may be able to act redundantly with MICU1, but attempts to transgenically rescue *MICU1* mutants by ectopic *MICU3* expression were unsuccessful. This result is consistent with a report showing that MICU3 binds to MICU1 but apparently enhances mitochondrial Ca^{2+} uptake (Patron et al., 2019).

In summary, we present a comprehensive analysis of the conserved components of the mitochondrial Ca^{2+} importer and its regulators. While loss of the various components results in dramatically different organismal phenotypes, ranging from the most severe deficit exemplified by the *MICU1* mutants to the mild consequences of mutating *MICU3*, such diverse phenotypes mirror the situation reported in humans so far. The first described patients with *MICU1* mutations exhibit a severe, complex neurological condition accompanied by muscular dystrophy and congenital myopathy, clearly associated with mitochondrial dysfunction (Logan et al., 2014), whereas a later study reported *MICU1* patients with a relatively mild fatigue syndrome (Lewis-Smith et al., 2016). One explanation for the reported phenotypic variability is that the consequence of perturbing mitochondrial Ca^{2+} uptake can be influenced by additional factors, the most obvious being genetic background. The genetic tools described here open up the possibility for a thorough analysis of the uniporter function in a powerful genetic model organism, which will advance our understanding of the role of mitochondrial Ca^{2+} in health and disease.

STAR★METHODS

Detailed methods are provided in the online version of this paper and include the following:

- KEY RESOURCES TABLE
- CONTACT FOR REAGENT AND RESOURCE SHARING
- EXPERIMENTAL MODEL AND SUBJECT DETAILS
- METHOD DETAILS
  - *Drosophila* mutagenesis
  - Generation of transgenic lines
  - Mitochondrial membrane potential and calcium flux
  - Locomotor and lifespan assays
  - Mitochondrial protein enrichment
  - Immunoblotting
  - Antibodies
  - Microscopy
  - Quantifying Mitochondrial Density
  - Axonal Transport
  - Respirometry analysis
  - ATP levels
  - RNA extraction, cDNA synthesis and qRT-PCR
- QUANTIFICATION AND STATISTICAL ANALYSIS

SUPPLEMENTAL INFORMATION

Supplemental Information can be found online at https://doi.org/10.1016/j.celrep.2019.04.033.

ACKNOWLEDGMENTS

This work is supported by MRC core funding (MC_UU_00015/4, MC-A070-5PSB0, and MC_UU_00015/6) and an ERC starting grant (DYNAMITO; 309742) to A.J.W., as well as by the Italian Ministry of Health "Ricerca Finalizzata" (GR-2011-02351151) to E.Z. T.P.G. and J.J.L. are supported by MRC studentships awarded via the MRC MBU. V.L.H. was funded by an EMBO Long-Term Fellowship (ALTF 740-2015) co-funded by the European Commission FP7 (Marie Curie Actions, LTFCOFUND2013, GA-2013-609409). Stocks were obtained from the Bloomington Drosophila Stock Center, which is supported by NIH grant P40OD018537, and material was obtained from the Drosophila Genomics Resource Center, which is supported by NIH grant 2P40OD010949. We thank Sarah Masood Alam and Ivana Giunta for technical help and all members of the Whitworth lab for discussions.

AUTHOR CONTRIBUTIONS

R.T., T.P.G., V.L.H., J.J.L., A.T.-F., and A.S.-M. designed and performed experiments and analyzed data. S.v.S. designed, performed, and analyzed the Ca^{2+} uptake assay with supervision from E.Z. R.T., T.P.G., and A.J.W. wrote the manuscript, with input from all authors. A.J.W. conceived the study, designed and performed experiments, analyzed data, and supervised the work.

DECLARATION OF INTERESTS

The authors declare no competing interests.

Received: November 20, 2018
Revised: March 21, 2019
Accepted: April 4, 2019
Published: April 30, 2019

REFERENCES

Antony, A.N., Paillard, M., Moffat, C., Juskeviciute, E., Correnti, J., Bolon, B., Rubin, E., Csordás, G., Seifert, E.L., Hoek, J.B., and Hajnóczky, G. (2016). MICU1 regulation of mitochondrial Ca(2+) uptake dictates survival and tissue regeneration. Nat. Commun. *7*, 10955.

Baldwin, K.R., Godena, V.K., Hewitt, V.L., and Whitworth, A.J. (2016). Axonal transport defects are a common phenotype in *Drosophila* models of ALS. Hum. Mol. Genet. *25*, 2378–2392.

Baughman, J.M., Perocchi, F., Girgis, H.S., Plovanich, M., Belcher-Timme, C.A., Sancak, Y., Bao, X.R., Strittmatter, L., Goldberger, O., Bogorad, R.L., et al. (2011). Integrative genomics identifies MCU as an essential component of the mitochondrial calcium uniporter. Nature *476*, 341–345.

Bick, A.G., Calvo, S.E., and Mootha, V.K. (2012). Evolutionary diversity of the mitochondrial calcium uniporter. Science *336*, 886.

Bischof, J., Björklund, M., Furger, E., Schertel, C., Taipale, J., and Basler, K. (2013). A versatile platform for creating a comprehensive UAS-ORFeome library in *Drosophila*. Development *140*, 2434–2442.

Choi, S., Quan, X., Bang, S., Yoo, H., Kim, J., Park, J., Park, K.S., and Chung, J. (2017). Mitochondrial calcium uniporter in *Drosophila* transfers calcium between the endoplasmic reticulum and mitochondria in oxidative stress-induced cell death. J. Biol. Chem. *292*, 14473–14485.

De Stefani, D., Raffaello, A., Teardo, E., Szabò, I., and Rizzuto, R. (2011). A forty-kilodalton protein of the inner membrane is the mitochondrial calcium uniporter. Nature *476*, 336–340.

Denton, R.M. (2009). Regulation of mitochondrial dehydrogenases by calcium ions. Biochim. Biophys. Acta *1787*, 1309–1316.

Edelstein, A.D., Tsuchida, M.A., Amodaj, N., Pinkard, H., Vale, R.D., and Stuurman, N. (2014). Advanced methods of microscope control using μManager software. J. Biol. Methods *1*, e11.

Gramates, L.S., Marygold, S.J., Santos, G.D., Urbano, J.M., Antonazzo, G., Matthews, B.B., Rey, A.J., Tabone, C.J., Crosby, M.A., Emmert, D.B., et al.; the FlyBase Consortium (2017). FlyBase at 25: looking to the future. Nucleic Acids Res. *45*, D663–D671.

Granatiero, V., De Stefani, D., and Rizzuto, R. (2017). Mitochondrial Calcium Handling in Physiology and Disease. Adv. Exp. Med. Biol. *982*, 25–47.

Gratz, S.J., Ukken, F.P., Rubinstein, C.D., Thiede, G., Donohue, L.K., Cummings, A.M., and O'Connor-Giles, K.M. (2014). Highly specific and efficient CRISPR/Cas9-catalyzed homology-directed repair in *Drosophila*. Genetics *196*, 961–971.

Graveley, B.R., Brooks, A.N., Carlson, J.W., Duff, M.O., Landolin, J.M., Yang, L., Artieri, C.G., van Baren, M.J., Boley, N., Booth, B.W., et al. (2011). The developmental transcriptome of *Drosophila melanogaster*. Nature *471*, 473–479.

Greene, J.C., Whitworth, A.J., Kuo, I., Andrews, L.A., Feany, M.B., and Pallanck, L.J. (2003). Mitochondrial pathology and apoptotic muscle degeneration in *Drosophila* parkin mutants. Proc. Natl. Acad. Sci. USA *100*, 4078–4083.

Harrington, J.L., and Murphy, E. (2015). The mitochondrial calcium uniporter: mice can live and die without it. J. Mol. Cell. Cardiol. *78*, 46–53.

Kamer, K.J., Grabarek, Z., and Mootha, V.K. (2017). High-affinity cooperative Ca^{2+} binding by MICU1-MICU2 serves as an on-off switch for the uniporter. EMBO Rep. *18*, 1397–1411.

Kamer, K.J., Sancak, Y., Fomina, Y., Meisel, J.D., Chaudhuri, D., Grabarek, Z., and Mootha, V.K. (2018). MICU1 imparts the mitochondrial uniporter with the ability to discriminate between Ca^{2+} and Mn^{2+}. Proc. Natl. Acad. Sci. USA *115*, E7960–E7969.

Kovács-Bogdán, E., Sancak, Y., Kamer, K.J., Plovanich, M., Jambhekar, A., Huber, R.J., Myre, M.A., Blower, M.D., and Mootha, V.K. (2014). Reconstitution of the mitochondrial calcium uniporter in yeast. Proc. Natl. Acad. Sci. USA *111*, 8985–8990.

Lee, J.J., Sanchez-Martinez, A., Zarate, A.M., Benincá, C., Mayor, U., Clague, M.J., and Whitworth, A.J. (2018). Basal mitophagy is widespread in *Drosophila* but minimally affected by loss of Pink1 or parkin. J. Cell Biol. *217*, 1613–1622.

Lewis-Smith, D., Kamer, K.J., Griffin, H., Childs, A.M., Pysden, K., Titov, D., Duff, J., Pyle, A., Taylor, R.W., Yu-Wai-Man, P., et al. (2016). Homozygous deletion in MICU1 presenting with fatigue and lethargy in childhood. Neurol. Genet. *2*, e59.

Liu, J.C., Liu, J., Holmström, K.M., Menazza, S., Parks, R.J., Fergusson, M.M., Yu, Z.X., Springer, D.A., Halsey, C., Liu, C., et al. (2016). MICU1 Serves as a Molecular Gatekeeper to Prevent *In Vivo* Mitochondrial Calcium Overload. Cell Rep. *16*, 1561–1573.

Liu, J.C., Parks, R.J., Liu, J., Stares, J., Rovira, I.I., Murphy, E., and Finkel, T. (2017). The *In Vivo* Biology of the Mitochondrial Calcium Uniporter. Adv. Exp. Med. Biol. *982*, 49–63.

Logan, C.V., Szabadkai, G., Sharpe, J.A., Parry, D.A., Torelli, S., Childs, A.M., Kriek, M., Phadke, R., Johnson, C.A., Roberts, N.Y., et al.; UK10K Consortium (2014). Loss-of-function mutations in MICU1 cause a brain and muscle disorder linked to primary alterations in mitochondrial calcium signaling. Nat. Genet. *46*, 188–193.

Luongo, T.S., Lambert, J.P., Yuan, A., Zhang, X., Gross, P., Song, J., Shanmughapriya, S., Gao, E., Jain, M., Houser, S.R., et al. (2015). The Mitochondrial Calcium Uniporter Matches Energetic Supply with Cardiac Workload during Stress and Modulates Permeability Transition. Cell Rep. *12*, 23–34.

Mallilankaraman, K., Doonan, P., Cárdenas, C., Chandramoorthy, H.C., Müller, M., Miller, R., Hoffman, N.E., Gandhirajan, R.K., Molgó, J., Birnbaum, M.J., et al. (2012). MICU1 is an essential gatekeeper for MCU-mediated mitochondrial Ca(2+) uptake that regulates cell survival. Cell *151*, 630–644.

Mammucari, C., Raffaello, A., Vecellio Reane, D., and Rizzuto, R. (2016). Molecular structure and pathophysiological roles of the Mitochondrial Calcium Uniporter. Biochim. Biophys. Acta *1863*, 2457–2464.

Murphy, E., Pan, X., Nguyen, T., Liu, J., Holmström, K.M., and Finkel, T. (2014). Unresolved questions from the analysis of mice lacking MCU expression. Biochem. Biophys. Res. Commun. *449*, 384–385.

Pan, X., Liu, J., Nguyen, T., Liu, C., Sun, J., Teng, Y., Fergusson, M.M., Rovira, I.I., Allen, M., Springer, D.A., et al. (2013). The physiological role of mitochondrial calcium revealed by mice lacking the mitochondrial calcium uniporter. Nat. Cell Biol. *15*, 1464–1472.

Patron, M., Granatiero, V., Espino, J., Rizzuto, R., and De Stefani, D. (2019). MICU3 is a tissue-specific enhancer of mitochondrial calcium uptake. Cell Death Differ. *26*, 179–195.

Perocchi, F., Gohil, V.M., Girgis, H.S., Bao, X.R., McCombs, J.E., Palmer, A.E., and Mootha, V.K. (2010). MICU1 encodes a mitochondrial EF hand protein required for Ca(2+) uptake. Nature *467*, 291–296.

Plovanich, M., Bogorad, R.L., Sancak, Y., Kamer, K.J., Strittmatter, L., Li, A.A., Girgis, H.S., Kuchimanchi, S., De Groot, J., Speciner, L., et al. (2013). MICU2, a paralog of MICU1, resides within the mitochondrial uniporter complex to regulate calcium handling. PLoS ONE *8*, e55785.

Pogson, J.H., Ivatt, R.M., Sanchez-Martinez, A., Tufi, R., Wilson, E., Mortiboys, H., and Whitworth, A.J. (2014). The complex I subunit NDUFA10 selectively rescues *Drosophila* pink1 mutants through a mechanism independent of mitophagy. PLoS Genet. *10*, e1004815.

Port, F., Chen, H.M., Lee, T., and Bullock, S.L. (2014). Optimized CRISPR/Cas tools for efficient germline and somatic genome engineering in *Drosophila*. Proc. Natl. Acad. Sci. USA *111*, E2967–E2976.

Raffaello, A., De Stefani, D., Sabbadin, D., Teardo, E., Merli, G., Picard, A., Checchetto, V., Moro, S., Szabò, I., and Rizzuto, R. (2013). The mitochondrial calcium uniporter is a multimer that can include a dominant-negative pore-forming subunit. EMBO J. *32*, 2362–2376.

Sancak, Y., Markhard, A.L., Kitami, T., Kovács-Bogdán, E., Kamer, K.J., Udeshi, N.D., Carr, S.A., Chaudhuri, D., Clapham, D.E., Li, A.A., et al. (2013). EMRE is an essential component of the mitochondrial calcium uniporter complex. Science *342*, 1379–1382.

Schindelin, J., Arganda-Carreras, I., Frise, E., Kaynig, V., Longair, M., Pietzsch, T., Preibisch, S., Rueden, C., Saalfeld, S., Schmid, B., et al. (2012). Fiji: an open-source platform for biological-image analysis. Nat. Methods *9*, 676–682.

Schmittgen, T.D., and Livak, K.J. (2008). Analyzing real-time PCR data by the comparative C(T) method. Nat. Protoc. *3*, 1101–1108.

von Stockum, S., Basso, E., Petronilli, V., Sabatelli, P., Forte, M.A., and Bernardi, P. (2011). Properties of Ca(2+) transport in mitochondria of *Drosophila melanogaster*. J. Biol. Chem. *286*, 41163–41170.

Wettmarshausen, J., Goh, V., Huang, K.T., Arduino, D.M., Tripathi, U., Leimpek, A., Cheng, Y., Pittis, A.A., Gabaldón, T., Mokranjac, D., et al. (2018). MICU1 Confers Protection from MCU-Dependent Manganese Toxicity. Cell Rep. *25*, 1425–1435.

Xu, S., and Chisholm, A.D. (2014). C. elegans epidermal wounding induces a mitochondrial ROS burst that promotes wound repair. Dev. Cell *31*, 48–60.

STAR★METHODS

KEY RESOURCES TABLE

REAGENT or RESOURCE	SOURCE	IDENTIFIER
Antibodies		
Mouse monoclonal anti-ATP5A	Abcam	RRID:AB_301447; Abcam: ab14748
Rabbit polyclonal anti-HA	Abcam	RRID:AB_307019; Abcam: ab9110
Mouse monoclonal anti-α-Tubulin, clone DM1A	Sigma	RRID:AB_477593; Sigma: T9026
Rabbit polyclonal anti-Porin	Millipore	Millipore PC548; RRID:AB_2257155
Mouse monoclonal anti-V5	Thermo Fisher Scientific	Thermo: R960-25; RRID:AB_2556564
Mouse monoclonal anti-Myc tag, clone 9B11	Cell Signaling	Cell Signaling: 2276; RRID:AB_331783
Goat polyclonal anti-Mouse IgG - H&L, HRP Conjugated	Abcam	Abcam: ab6789, RRID:AB_955439
Goat polyclonal anti-Rabbit IgG (H+L) Cross-Adsorbed Secondary Antibody, HRP	Thermo Fisher Scientific	Invitrogen G21234; RRID:AB_2536530
Goat polyclonal anti-Mouse IgG (H+L) Cross-Adsorbed Secondary Antibody, Alexa Fluor 488	Abcam	Thermo: A-11001; RRID:AB_2534069
Rabbit polyclonal anti-MCU (targeting RTQENTPPTLTE EKAERKY)	Pepceuticals	N/A
Chemicals, peptides, and recombinant proteins		
NotI FastDigest	Thermo Fisher Scientific	Thermo: FD0593
XhoI FastDigest	Thermo Fisher Scientific	Thermo: FD0694
XbaI FastDigest	Thermo Fisher Scientific	Thermo: FD0684
EcoRI FastDigest	Thermo Fisher Scientific	Thermo: FD0274
Bovine Serum Albumin	Sigma	Sigma: A7030
Mannitol	Sigma	Sigma: M9546
Sucrose	Sigma	Sigma: S9378
HEPES	Sigma	Sigma: H3375
EGTA	Sigma	Sigma: E0396
Taurine	Sigma	Sigma: T0625
Triton X-100	Sigma	Sigma: T8787
Tween 20	Thermo Fisher Scientific	Thermo: BP337500
cOmplete, Mini, EDTA-free Protease Inhibitor Cocktail	Sigma	Roche: 4693159001
2-Mercaptoethanol	Sigma	Sigma: M6250
$MgCl_2$	Sigma	Sigma: M8266
ECL-Prime	Sigma	Sigma: GERPN2232
4% Formaldehyde	Thermo Fisher Scientific	Thermo: 28908
Prolong Diamond Antifade Mounting Medium	Thermo Fisher Scientific	Thermo: P36965
Sylguard	Sigma	Sigma: 761028
Malate	Sigma	Sigma: M 1000
Glutamate	Sigma	Sigma: G 1626
Succinate	Sigma	Sigma: S 2378
ADP	Sigma	Sigma: A 5285
Rotenone	Sigma	Sigma: R 8875
Critical commercial assays		
CellTiter-Glo Luminescent Cell Viability Assay	Promega	Promega: G7570
RNeasy RNA purification kit	QIAGEN	QIAGEN: 74106
ProtoScript® II first strand cDNA Synthesis Kit	New England BioLabs	NEB: E6560S
DC Protein Assay Kit	Bio-Rad	N/A

(*Continued on next page*)

Continued

REAGENT or RESOURCE	SOURCE	IDENTIFIER
iQ SYBR® Green Supermix	Bio-Rad	170-8880
Turbo DNase Free	Ambion	AM1907
Experimental Models: organisms/strains		
D. melanogaster: w[1118]	BDSC (RRID:SCR_006457)	RRID:BDSC_6326
D. melanogaster: y[1] w[67c23]; P{w[+mC] y[+mDint2] = EPgy2}MCU[EY01803]	BDSC	RRID:BDSC_16357
D. melanogaster: y[1] w[67c23]; P{y[+mDint2] w[BR.E.BR] = SUPor-P}MICU1[KG04119]	BDSC	RRID:BDSC_13588
D. melanogaster: y[1] M{w[+mC] = nos-Cas9.P}ZH-2A w[*]	BDSC	RRID: BDSC_54591
D. melanogaster: w[*]; Kr[If-1]/CyO; P{w[+mW.hs] = GAL4-da.G32}UH1	BDSC	RRID:BDSC_55850
D. melanogaster: w[*]; P{w[+mW.hs] = GAL4-arm.S}11	BDSC	RRID:BDSC_1561
D. melanogaster: w[*]; P{w[+mC] = GAL4-ninaE.GMR}12	BDSC	RRID:BDSC_1104
D. melanogaster: y[1] w[*]; P{w[+mC] = CCAP-GAL4.P}16/CyO	BDSC	RRID:BDSC_25685
D. melanogaster: w[*]; P{w[+mW.hs] = GawB}tey [5053A]/TM6B, Tb[+]	BDSC	RRID:BDSC_2702
D. melanogaster: w[1118]; P{w[+mC] = UAS-mito-HA-GFP.AP}2/CyO	BDSC	RRID:BDSC_8442
D. melanogaster: w[1118]; P{w[+mC] = UAS-mito-HA-GFP.AP}3, e[1]	BDSC	RRID:BDSC_8443
D. melanogaster: y[*] w[*];P{w[+mC] = UAS-tdTomato.mito}1	BDSC	RRID:DGGR_117015
D. melanogaster: M{UAS-MICU1.ORF.3xHA}ZH-86Fb	FlyORF	FlyORF Line ID: F000962
Oligonucleotides		
MCU[1] Forward Primer: GCAACTTCAGCATATGACC	This paper	N/A
MCU[1] Reverse Primer: GGAATTGGGATGCCATAGC	This paper	N/A
EMRE[1] Forward Primer: GCGCTTTTCAACACTACTAC	This paper	N/A
EMRE[1] Reverse Primer: GGTATGACGGCACAGAAGATG	This paper	N/A
MICU3[27] Forward Primer: CTCGATCTCTGATCCCGCA	This paper	N/A
MICU3[27] Reverse Primer: TCGTGCAGAAAACAACTACATTT	This paper	N/A
qPCR Primers	This paper (See Table S1)	N/A
Recombinant DNA		
pCFD4	Addgene	Addgene: 49411
pUAST-attB	Drosophila Genomics Resource Centre	DGRC: 1419
LD26402	Drosophila Genomics Resource Centre	DGRC: 2351
IP17639	Drosophila Genomics Resource Centre	DGRC: 1605128
LD23951	Drosophila Genomics Resource Centre	DGRC: 7119
RH09265	Drosophila Genomics Resource Centre	DGRC: 1153329
Software and algorithms		
Datlab	Oroboros	N/A
Prism v7	GraphPad	RRID:SCR_002798
Fiji	Fiji.sc	RRID:SCR_002285
Micro-Manager	Micro-manager.org	RRID:SCR_016865
Other		
Microinjection Services	BestGene Inc., or Department of Genetics, University of Cambridge	N/A

CONTACT FOR REAGENT AND RESOURCE SHARING

Further information and requests for resources and reagents should be directed to and will be fulfilled by the Lead Contact, Alexander J. Whitworth (a.whitworth@mrc-mbu.cam.ac.uk).

EXPERIMENTAL MODEL AND SUBJECT DETAILS

Flies were raised under standard conditions at 25°C on food consisting of agar, cornmeal, molasses, propionic acid and yeast in a 12h:12h light:dark cycle. The following strains were obtained from the Bloomington *Drosophila* Stock Center (RRID:SCR_006457): *w*^{1118} (RRID:BDSC_6326), *MCU*^{EY08610} (RRID:BDSC_16357), *MICU1*^{KG04119} (RRID:BDSC_13588), *da-GAL4* (RRID:BDSC_55850), *arm-GAL4* (RRID:BDSC_1561), *GMR-GAL4* (RRID:BDSC_1104), *CCAP-GAL4* (RRID:BDSC_25685), *M12-GAL4* (RRID:BDSC_2702), *UAS-mito-HA-GFP* (RRID:BDSC_8442, RRID:BDSC_8443), *UAS-mito.tdTomato* (RRID:DGGR_117015). *UAS-MICU1-A-HA* (F000962) was obtained from the FlyORF collection (Bischof et al., 2013). Experimental procedures and fly husbandry were performed in accordance with local Biological Services committee approval.

METHOD DETAILS

Drosophila mutagenesis

Mobilisation of the *MCU*^{EY08610} and *MICU1*^{KG04119} transposable elements was used to generate *MCU* (*CG18769*) and *MICU1* (*CG4495*) mutants, termed *MCU*^{1} and *MICU1*^{32}. Several precise excisions of the P-elements (revertants) were also recovered. For *EMRE* (*CG17680*) and *MICU3* (*CG4662*), a CRISPR-based strategy was employed. Here, guide RNAs (gRNAs) were evaluated using an online tool (http://targetfinder.flycrispr.neuro.brown.edu/) (Gratz et al., 2014). Where possible, gRNAs toward the 5′ end of the protein coding sequence without predicted off-targets were selected. gRNAs were cloned into the pCFD4 vector (Addgene 49411), and the resulting constructs were verified by sequencing before being sent for transgenesis by phiC31 site-directed integration into attP40 and attP2 sites (BestGene Inc., or Department of Genetics, University of Cambridge). These gRNA-expressing flies were crossed to *y*^{1} M{*nos-cas9*, *w*$^{+}$} ZH-2A *w** (RRID: BDSC_54591) for mutagenesis. Mutant lines were screened via sequencing of PCR products spanning the targeted gene region. The mapped breakpoints for each mutant are as follows: *MCU*^{1}, 3L:6550718-6552274; *EMRE*^{1}, 2R:18147830-18147821, 2R:18147812-18147804; *EMRE*^{2}, 2R: 18147814-18147795; *EMRE*^{3}, 2R:18147805-18147805; *MICU1*^{32}, 2L:7173086-7184065; *MICU3*^{27}, 3R:19850278-19850278. All genomic coordinates are according to FlyBase/BDGP Release 6 (Gramates et al., 2017). All the mutants lines used in this study were backcrossed to an isogenic *w*^{1118} strain (RRID:BDSC_6326), for 4-6 generations before use.

Generation of transgenic lines

The following transgenes were generated by cloning into the pUAST-attB vector (BestGene Inc.).

UAS-MCU

MCU was amplified from cDNA clone LD26402 (equivalent to isoforms A, B, C and D) and inserted between the *Not*I and *Xho*I sites. *UAS-MICU1-B-HA*: *MICU1-B* was amplified from cDNA IP17639 to include a single 3′ HA tag, and was inserted between the *Not*I and *Xba*I sites. *UAS-EMRE-myc*: *EMRE* was amplified from genomic DNA with primers encoding a single 3′ Myc tag, and was inserted between the *EcoR*I and *Xho*I sites. *UAS-MICU3-A-V5*, *UAS-MICU3-C-V5*: *MICU3-A* and *MICU3-C* were amplified from cDNA clones lD23951 and RH09265 respectively, including a single 3′ V5 tag, and were inserted between the *Not*I and *Xba*I sites.

All cDNA clones were obtained from *Drosophila* Genomics Resource Center (Bloomington, Indiana). Constructs were verified by sequencing before being sent for transgenesis by phiC31 site-directed integration into attP40 and attP2 sites (BestGene Inc., or Department of Genetics, University of Cambridge). For all integration events, multiple independent lines were initially isolated, verified by PCR and assessed for consistent effects before selecting a single line of each integration site for further study.

Mitochondrial membrane potential and calcium flux

Mitochondria were prepared from ~50 whole adult flies by differential centrifugation. Samples were homogenized with a Dounce glass potter and a loose-fitting glass pestle in a mannitol-sucrose buffer (225 mM mannitol, 75 mM sucrose, 5 mM HEPES, 0.1 mM EGTA, pH 7.4) supplemented with 2% BSA. Samples were centrifuged at 1,500 × *g* at 4°C for 6 min. The supernatant was filtered through a fine mesh, and centrifuged at 7,000 × *g* at 4°C for 6 min. The resulting pellet was resuspended in mannitol-sucrose buffer without BSA before being centrifuged at 7,000 x *g* under the same conditions as above and resuspended in a small volume (~50 µL) of mannitol-sucrose buffer. Protein concentration was measured using the Biuret test.

Mitochondrial membrane potential of isolated mitochondria was measured based on the fluorescence quenching of Rhodamine123 (Rh123; Molecular Probes) and mitochondrial Ca^{2+} fluxes were measured by Calcium Green 5N (Molecular Probes) fluorescence at 25°C (von Stockum et al., 2011) using a Fluoroskan Ascent FL (Thermo Electron) plate reader (excitation and emission wavelengths of 485 and 538 nm, respectively with a 10 nm bandpass filter) at a mitochondrial concentration of 1 mg/mL. The incubation medium contained 250 mM sucrose, 10 mM MOPS-Tris, 5 mM/2.5 mM glutamate/malate-Tris, 5 mM Pi-Tris, 10 µM EGTA, and 0.4 µM Rhodamine123, or 0.5 µM Calcium Green 5N, pH 7.4. Addition of Ruthenium Red (RuR, 2 µM) was made directly into the well containing the assay medium before mitochondria were added. Further additions were made as indicated in the figure legends.

Locomotor and lifespan assays

Climbing (negative geotaxis assay) was assessed as previously described with minor modifications (Greene et al., 2003). Briefly, for climbing, 20-25 males were placed into the first chamber of a 'Benzer' counter-current apparatus, tapped to the bottom, and given 10 s to climb a 10 cm distance. This procedure was repeated five times, and the number of flies remaining in each chamber was counted. The weighted performance of several group of flies for each genotype was normalized to the maximum possible score and expressed as Climbing Index.

For larval locomotion (crawling), mid-third instar larvae were picked from the surface of the food using a brush and cleaned using a moist Kimwipe. Excessive handling was minimized at all stages. Larvae were assayed individually by placing onto plates containing freshly set 1% agarose, where they acclimatised for 30 s, after which peristaltic waves were counted for one minute. Larvae of different genotypes were placed onto different plates, and no more than 6 larvae were assayed for any individual plate.

For lifespan experiments, flies were grown under identical conditions at low-density. Groups of approximately 20-25 males of each genotype were collected under very light anesthesia, placed into separate vials with food and maintained at 25°C. Flies were transferred into vials containing fresh food every 2-3 days, and the number of dead flies was recorded. Percent survival was calculated at the end of the experiment after correcting for any loss resulting from handling.

Mitochondrial protein enrichment

Crude mitochondrial extracts were obtained from approximately 100 flies per sample. After 5 min on ice, flies were homogenized in a glass tissue grinder containing 2 mL of cold mitochondrial isotonic buffer (225 mM mannitol, 75 mM sucrose, 5 mM HEPES, 0.5 mM EGTA, 2 mM taurine, pH 7.25) for 30-60 s until uniform. Subsequently, the homogenates were centrifuged at 500 × *g* at 4°C for 5 min. The resulting supernatants were passed through a 100 μm nylon sieve (Cell Strainer REF 352360, BD Falcon, USA**)**, centrifuged at 11,000 × *g* for 10 min at 4°C, and pellets were stored at −80°C until use. The final mitochondrial pellets were subsequently used for lysis in 50 μL of RIPA buffer (50 mM Tris-HCl, pH 8.0; 150 mM NaCl; 1 mM EDTA, 0.5% SDS, 1% (vol/vol) Triton X-100) with cOmplete mini EDTA-free protease inhibitors (Roche) for 10 min on ice. After carrying out three freeze-thaw cycles with dry ice and a 37°C water bath, the lysates were centrifuged at 20,000 × *g* for 5 min and the supernatants taken for SDS-PAGE.

Immunoblotting

For MCU and EMRE expression analysis, mitochondrial proteins were isolated from whole adult flies according to the method described in the previous section. For MICU1 and MICU3 overexpression analysis flies were homogenized in a PBS-based lysis buffer with lithium dodecyl sulfate containing β-Mercaptoethanol and supplemented with cOmplete mini EDTA-free protease inhibitors (Roche). Equivalent amounts of proteins were resolved by SDS-PAGE and transferred onto nitrocellulose membrane using a semi-dry Transblot apparatus (BioRad) according to the manufacturer's instructions. The membranes were blocked in TBST (0.15 M NaCl and 10 mM Tris-HCl; pH 7.5, 0.1% Tween 20) containing 5% (w/v) dried non-fat milk (blocking solution) for 1 h at room temperature and probed with the indicated primary antibody before being incubated with the appropriate HRP-conjugated secondary antibody. Antibody complexes were visualized by an ECL-Prime enhanced chemiluminescence detection kit (Amersham) using a ChemiDoc XRS+ molecular imager (BioRad).

Antibodies

For immunoblot experiments, the following antibodies were used: mouse anti-ATP5A (Abcam ab14748; RRID:AB_301447; 1:20000), rabbit anti-HA (Abcam ab9110; RRID:AB_307019; 1:1000), mouse anti-α-Tubulin (Sigma T9026, clone DM1A; RRID:AB_477593; 1:1500), rabbit anti-Porin (Millipore PC548; RRID:AB_2257155; 1:5000), mouse anti-V5 (Thermo Fisher Scientific R960-25; RRID:AB_2556564; 1:2000), mouse anti-Myc tag (Cell Signaling, clone 9B11; RRID:AB_331783; 1:800). Horseradish peroxidase-conjugated secondary antibodies: anti-mouse (Abcam ab6789-1; RRID:AB_955439; 1:5000-1:40000), anti-rabbit (Invitrogen G21234; RRID:AB_2536530; 1:3000 to 1:5000). Anti-MCU antiserum was raised in rabbits against a KLH-conjugated C-terminal peptide, RTQENTPPTLTEEKAERKY (Pepceuticals, 1:1000). For immunohistochemistry, tissues were incubated with mouse anti-ATP5A (Abcam ab14748; RRID:AB_301447; 1:500) and secondary antibody anti-mouse AF488 (Invitrogen: A11001; RRID:AB_2534069; 1:200).

Microscopy

Indirect flight muscle was dissected and fixed in 4% formaldehyde (Agar scientific; R1926) in PBS for 30 minutes, washed twice with PBS, and mounted on slides in Prolong Diamond Antifade mounting medium (Thermo Fisher Scientific; RRID:SCR_015961). Larval epidermal cells were prepared as previously described (Lee et al., 2018). Larvae were dissected in PBS and fixed in 4% formaldehyde, for 30 min, permeabilized in 0.3% Triton X-100 for 30 min, and blocked with 0.3% Triton X-100 plus 1% bovine serum albumin in PBS for 1 h at room temperature. Tissues were incubated with anti-ATP5A antibody diluted in 0.3% Triton X-100 plus 1% bovine serum albumin in PBS overnight at 4°C, rinsed three times 10 min with 0.3% Triton X-100 in PBS, and incubated with the appropriate fluorescent secondary antibodies for 2 h at room temperature. The tissues were washed twice in PBS and mounted on slides using Prolong Diamond Antifade mounting medium (Thermo Fisher Scientific). Fluorescence imaging was conducted with a Zeiss LSM 880 confocal microscope/Nikon Plan-Apochromat 63x/1.4 NA oil immersion objective. For adult eyes, images were acquired using a Leica DFC490 camera mounted on a Leica MZ6 stereomicroscope set at maximum zoom.

Quantifying Mitochondrial Density

Images of larval epidermal cells we acquired as above and processed in Fiji software (Schindelin et al., 2012). Briefly, images encompassing several cells were acquired using a 63x/1.4 NA oil immersion objective. Several 15 μm^2 regions, avoiding especially sparse or dense areas, were selected. Exposure was adjusted using the Threshold function, selecting Otsu type, B&W and Auto. Images were made binary, and Analyze Particles function was applied (size: 0.1-infinity).

Axonal Transport

Analysis of axonal transport was performed on wandering third instar larvae as previously described (Baldwin et al., 2016). Larvae were pinned at each end dorsal side up to a Sylgard (Sigma 761028) slide and cut along the dorsal midline using micro-dissection scissors. Larvae were covered in dissection solution (128 mM NaCl, 1 mM EGTA, 4 mM $MgCl_2$, 2 mM KCl, 5 mM HEPES and 36 mM sucrose, adjusted to pH 7 using NaOH), the sides were pinned back and the internal organs removed. Movies were taken using a Nikon E800 microscope with a 60x water immersion lens (NA 1.0 Nikon Fluor WD 2.0) and an LED light source driven by Micro-Manager 1.4.22 Freeware (Edelstein et al., 2014). A CMOS camera (01-OPTIMOS-F-M-16-C) was used to record 100 frames at a rate of 1 frame per 5 s for *CCAP-GAL4* samples or 1 frame per 2.5 s for *M12-GAL4* samples. Movies were converted into kymographs using Fiji (Schindelin et al., 2012) and quantified manually.

Respirometry analysis

Respiration was monitored at 30°C using an Oxygraph-2k high-resolution respirometer (OROBOROS Instruments) using a chamber volume set to 2 mL. Calibration with air-saturated medium was performed daily. Data acquisition and analysis were carried out using Datlab software (OROBOROS Instruments). Five flies per genotype (equal weight) were homogenized in respiration buffer (120 mM sucrose, 50 mM KCl, 20 mM Tris-HCl, 4 mM KH_2PO_4, 2 mM $MgCl_2$, and 1 mM EGTA, 1 g/l fatty acid-free BSA, pH 7.2). For *MICU3*^{27} experiments, 5 heads per genotype were used. For coupled (state 3) assays, complex I-linked respiration was measured at saturating concentrations of malate (2 mM), glutamate (10 mM) and adenosine diphosphate (ADP, 2.5 mM). Complex II-linked respiration was assayed in respiration buffer supplemented with 0.15 μM rotenone, 10 mM succinate and 2.5 mM ADP.

ATP levels

The ATP assay was performed as described previously (Pogson et al., 2014). Briefly, five male flies of the indicated age or 10 larvae for each genotype were homogenized in 100 μL 6 M guanidine-Tris/EDTA extraction buffer and subjected to rapid freezing in liquid nitrogen. Homogenates were diluted 1/100 with the extraction buffer and mixed with the luminescent solution (CellTiter-Glo Luminescent Cell Viability Assay, Promega). Luminescence was measured with a SpectraMax Gemini XPS luminometer (Molecular Devices). The average luminescent signal from technical triplicates was expressed relative to protein levels, quantified using the DC Protein Assay kit (Bio-Rad). Data from 2-4 independent experiments were averaged and the luminescence expressed as a percentage of the control.

RNA extraction, cDNA synthesis and qRT-PCR

Isolation of total RNA was performed using the RNeasy RNA purification kit (QIAGEN); cDNA was synthesized from total RNA using ProtoScript® II first strand cDNA Synthesis Kit (New England BioLabs, E6560S) according to manufacturer's instructions. Total RNA concentration was ascertained spectrophotometrically, and equivalent amounts of total RNA underwent reverse transcription for each sample. Quantitative real-time PCR (qRT-PCR) was performed on a CFX96 Touch Real-Time PCR Detection System. Gene-specific primers were designed to have oligos spanning an intron whenever possible. Primer sequences (summarized in Table S1) were as follows: *EMRE* forward 5′-ACATGTCCAGCGTGTACTTTC-3′ and reverse 5′-GGTATGACGGCACAGAAGATG-3′; *MICU1* forward 5′-GTGGCCATGGTCAATCTTTC-3′ and reverse 5′-TTGTTGCTGAGTTGGTTGTCA-3′; *MICU3* forward 5-GATCCACAAAC CAAGCGAAT-3′ and reverse 5′-CCTCTTCCGGCTCTTGCT-3′; *sulfateless* forward 5′-AAGCTGTCGATTTGAGTAGCAA-3′ and reverse 5′-GACTGTCCACTCGCAATCAG-3′; *javelin* forward 5′-GCGGATTTTTCCGTGAATC-3′ and reverse 5′-TCTGGCTCTGGGT GTCATC-3′; and *RpL32* forward 5′-GCCGCTTCAAGGGACAGTATCTG-3′ and reverse 5′-AAACGCGGTTCTGCATGAG-3′. Carry-over DNA was removed with Turbo DNase free (Ambion, Cat. No. AM1907) according to the manufacturer's protocol. The relative transcript levels of each target gene were normalized against *RpL32* mRNA levels; quantification was performed using the comparative C_T method (Schmittgen and Livak, 2008).

QUANTIFICATION AND STATISTICAL ANALYSIS

Data are reported as mean ± SD, SEM or 95% confidence interval (CI) as indicated in figure legends. For climbing analysis, Kruskal-Wallis non-parametric test with Dunn's post hoc correction for multiple comparisons was used. For lifespan experiments, significance levels were determined by log-rank tests and reported in the figure legends. Mitochondrial transport was analyzed by one-way ANOVA, and larval crawling, ATP, and Oroboros measurements analyzed by two-tailed unpaired t test. Unless specifically indicated, no significant difference was found between a sample and any other sample in the analysis. Where n is indicated in the figure legends, these refer to biological replicates or number of animals tested. No data points were excluded from the analyses. Analyses were performed using GraphPad Prism 7 software (RRID:SCR_002798).

Cell Reports, Volume *27*

Supplemental Information

Comprehensive Genetic Characterization of Mitochondrial Ca^{2+} Uniporter Components Reveals Their Different Physiological Requirements *In Vivo*

Roberta Tufi, Thomas P. Gleeson, Sophia von Stockum, Victoria L. Hewitt, Juliette J. Lee, Ana Terriente-Felix, Alvaro Sanchez-Martinez, Elena Ziviani, and Alexander J. Whitworth

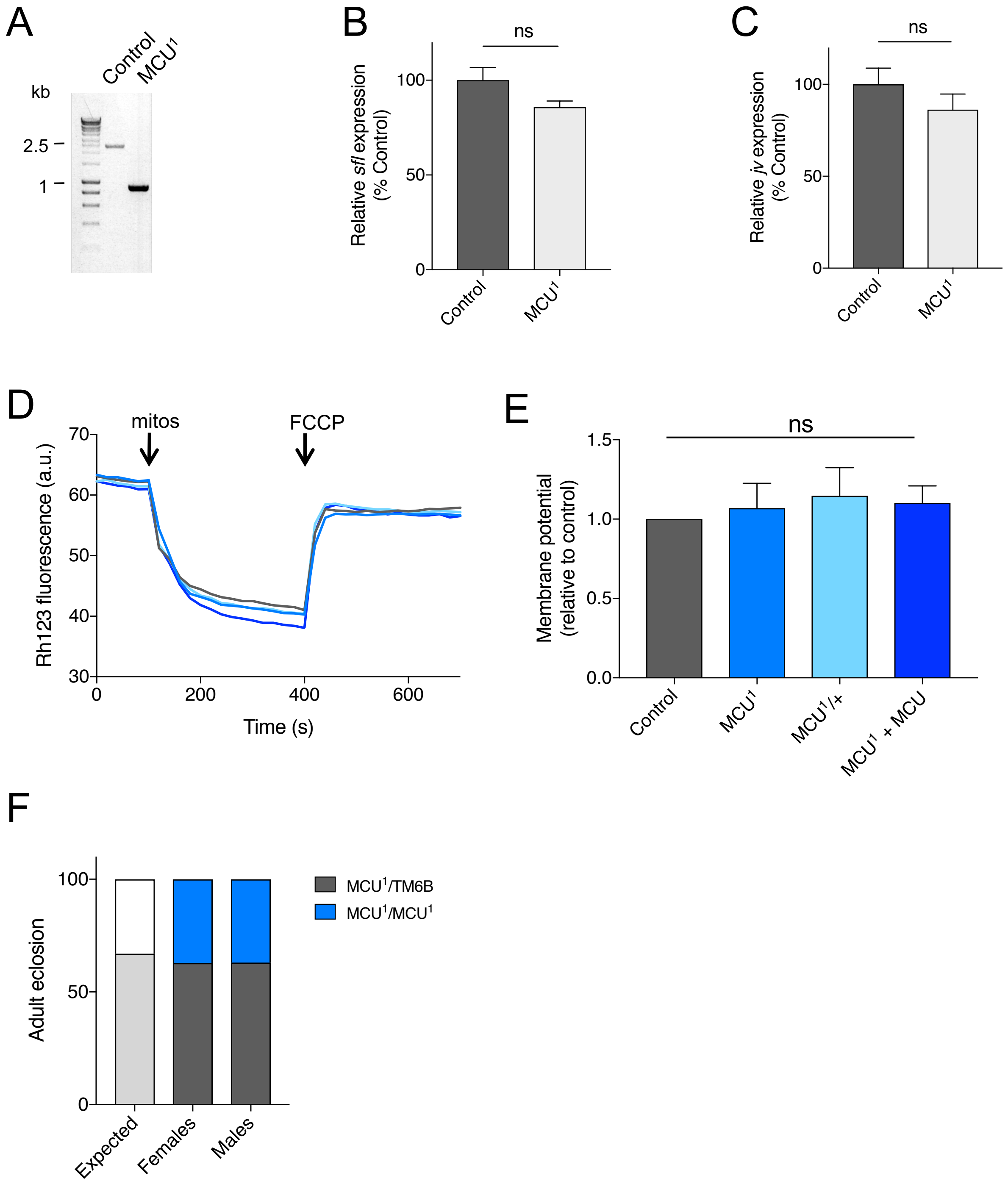

Figure S1. Additional characterisation of *MCU*[1] mutants. Related to Figure 1.

A. DNA analysis of *MCU*[1]. Primer sequences are detailed in the 'STAR Methods' section. The control genotype yielded a ~2.5 kb band, compared to ~900 bp for *MCU*[1] homozygotes.

B, C. Relative transcript level of *sfl* (**B**) or *jv* (**C**) for control and *MCU*[1] flies (mean ± SD; n = 3).

D. Representative traces of Rhodamine 123 (Rh123) fluorescence (a.u.: arbitrary units) in isolated mitochondria of the indicated genotypes used for the Ca^{2+} uptake assays for *MCU* (with *da-GAL4* expressed *UAS-MCU*).

E. Relative membrane potential was determined through Rh123 fluorescence change before and after addition of FCCP (ΔF-F0) normalized to controls (mean ± SEM; n = 3).

F. The percentage of adult flies eclosing as homozygous *MCU*[1] mutants *versus* balanced heterozygotes, together with the expected Mendelian ratio in the offspring (n > 700).

The control genotype is w^{1118}.

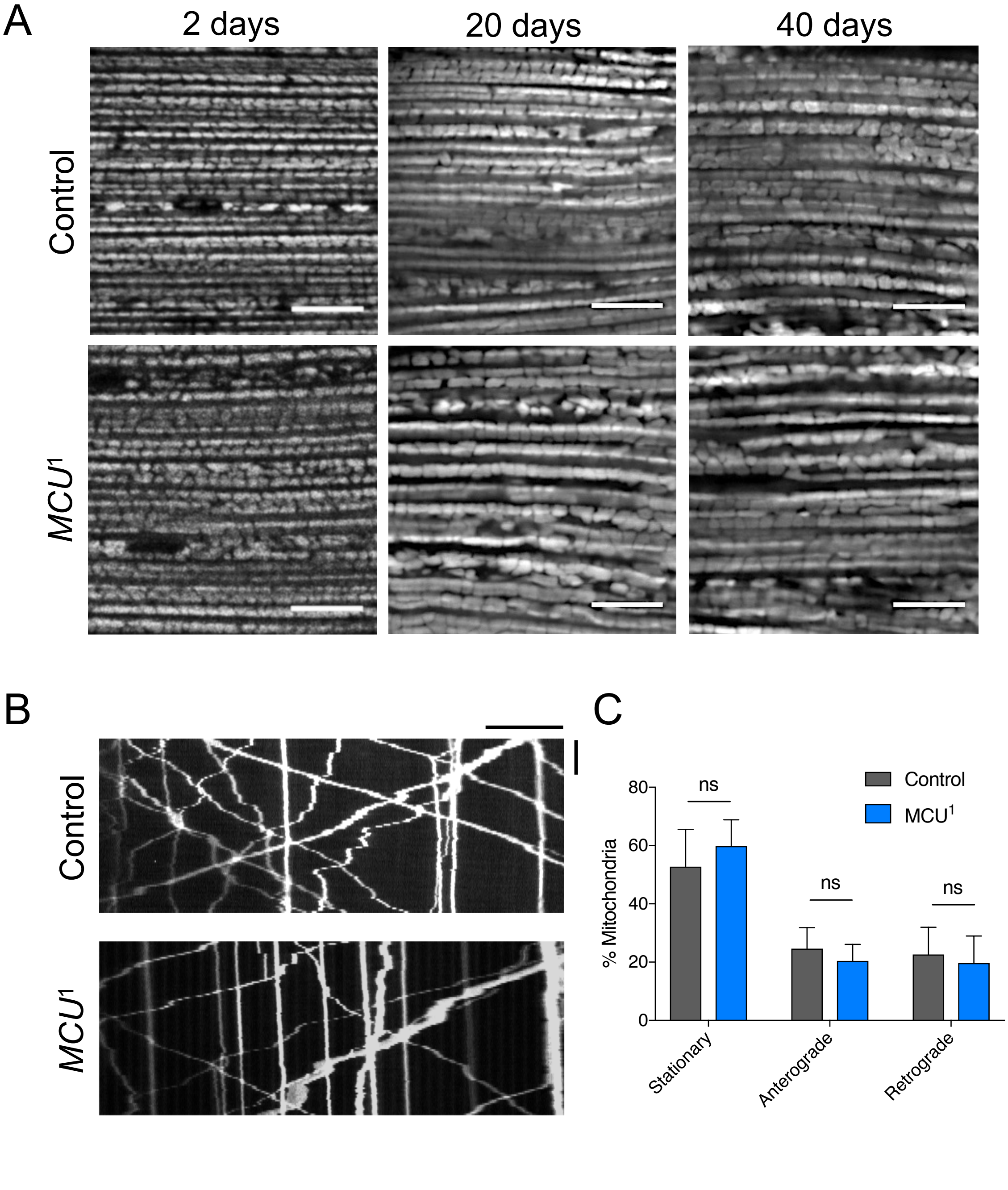

Figure S2. Mitochondrial morphology and dynamics in *MCU*^{1} mutants. Related to Figure 1.

A. Mitochondrial morphology of control or *MCU*^{1} adult indirect flight muscle at 2, 20, or 40 days post-eclosion. Scale bar = 10 µm. Genotypes - <u>Control</u>: *UAS-mito-HA-GFP/+; da-GAL4/+*. <u>*MCU*^{1}</u>: *UAS-mito-HA-GFP/+; MCU*^{1}*/MCU*^{1}*, da-GAL4*.

B. Representative kymographs of mitochondrial axonal transport in control and *MCU*^{1} larvae. Scale bars: horizontal = 20 µm, vertical = 100 s. Genotypes - <u>Control</u>: *CCAP-GAL4, UAS-mito.tdTomato/+*. <u>*MCU*^{1}</u>: *CCAP-GAL4, UAS-mito.tdTomato/+; MCU*^{1}*/MCU*^{1}.

C. Quantification of mitochondrial transport shown in B. Statistical analysis: one-way ANOVA (mean ± 95% CI; n = 10; ns, non-significant).

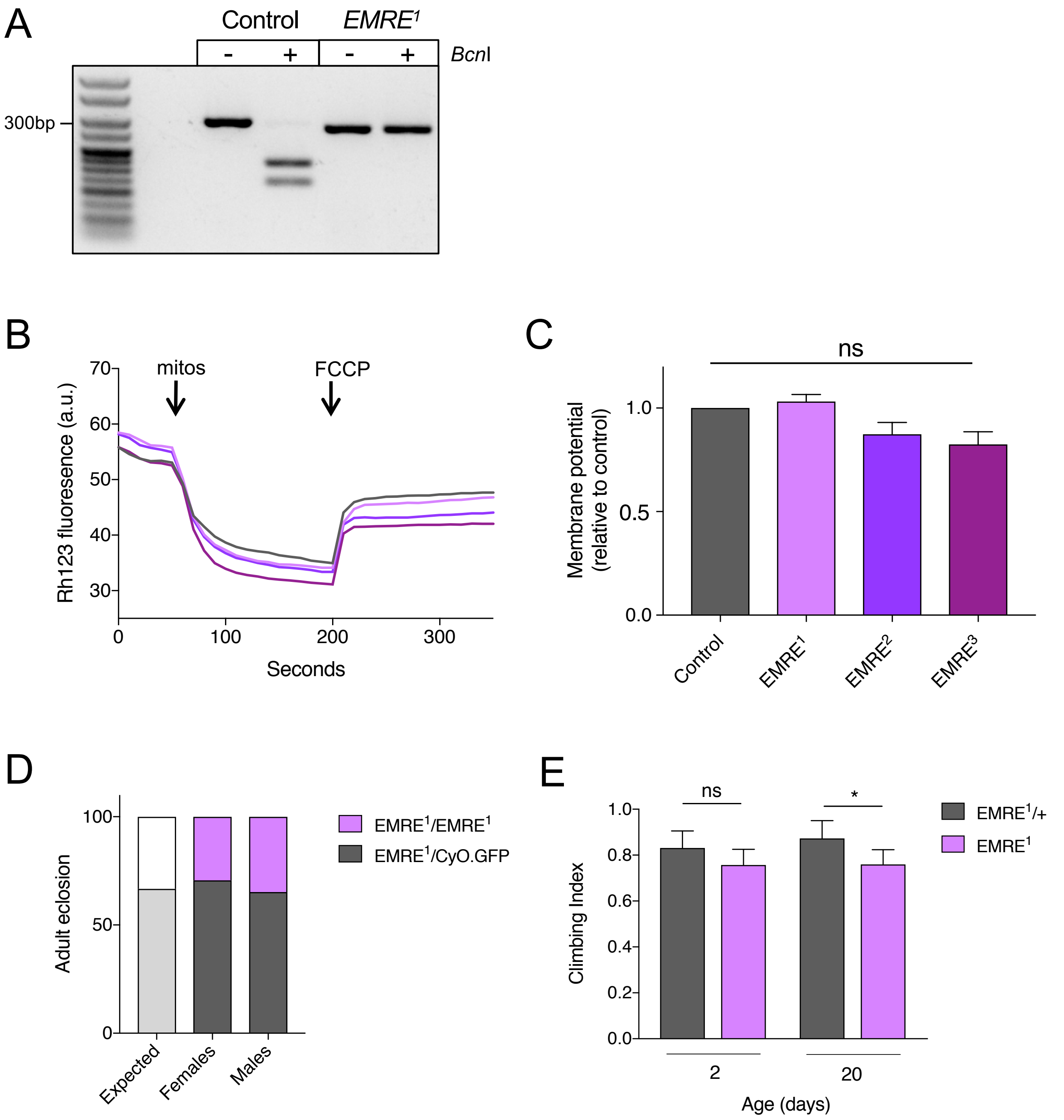

Figure S3. Additional characterisation of *EMRE*[1] mutants. Related to Figure 2.
A. RFLP analysis of control and *EMRE*[1] PCR products with *Bcn*I.
B. Representative traces of Rhodamine 123 (Rh123) fluorescence (a.u.: arbitrary units) in isolated mitochondria of the indicated genotypes used for the Ca^{2+} uptake assays for *EMRE* mutants.
C. Relative membrane potential was determined through Rh123 fluorescence change before and after addition of FCCP (ΔF-F0) normalized to controls (mean ± SEM; n = 3).
D. The percentage of adult flies eclosing as homozygous *EMRE*[1] mutants *versus* balanced heterozygotes, together with the expected Mendelian ratio in the offspring (n > 360).
The control genotype is w^{1118}.
E. Climbing assay of *EMRE*[1]/+ and *EMRE*[1] flies at 2 and 20 days post-eclosion. Statistical analysis: Kruskal-Wallis test with Dunn's post-hoc correction for multiple comparisons (mean ± 95% CI; n > 50; * $P < 0.05$, ns, non-significant).

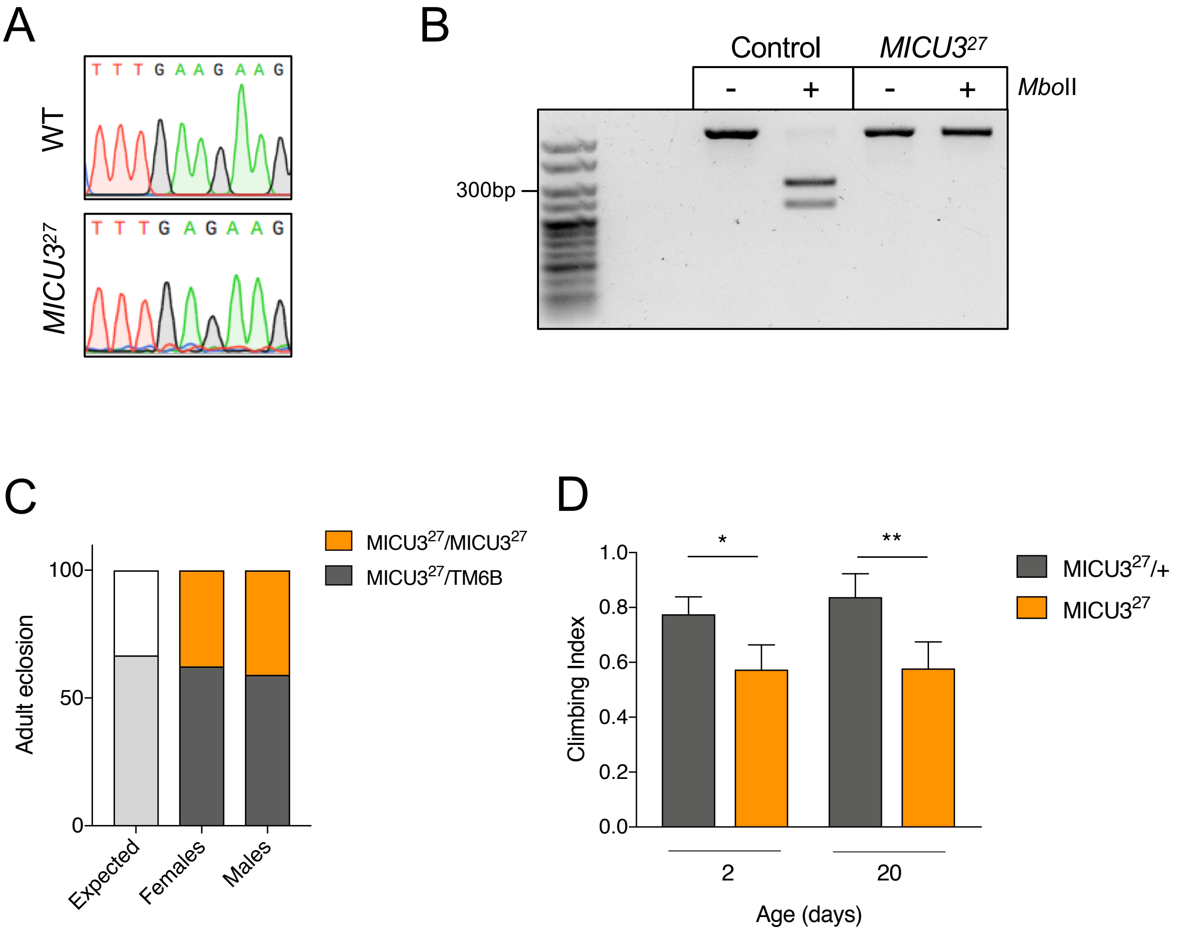

Figure S4. Additional characterisation of *MICU3*^{27} mutants. Related to Figure 4.
A. Representative sequencing chromatograms for wild-type and *MICU3*^{27} showing the single base deletion of *MICU3*^{27}.
B. RFLP analysis of control and *MICU3*^{27} PCR products with *Mbo*II.
C. The percentage of adult flies eclosing as homozygous *MICU3*^{27} mutants *versus* balanced heterozygotes, together with the expected Mendelian ratio in the offspring (n > 225).
The control genotype is w^{1118}.
D. Climbing assay of *MICU3*^{27}/+ and *MICU3*^{27} flies at 2 and 20 days post-eclosion. Statistical analysis: Kruskal-Wallis test with Dunn's post-hoc correction for multiple comparisons (mean ± 95% CI; n > 40; * $P < 0.05$, ** $P < 0.01$).

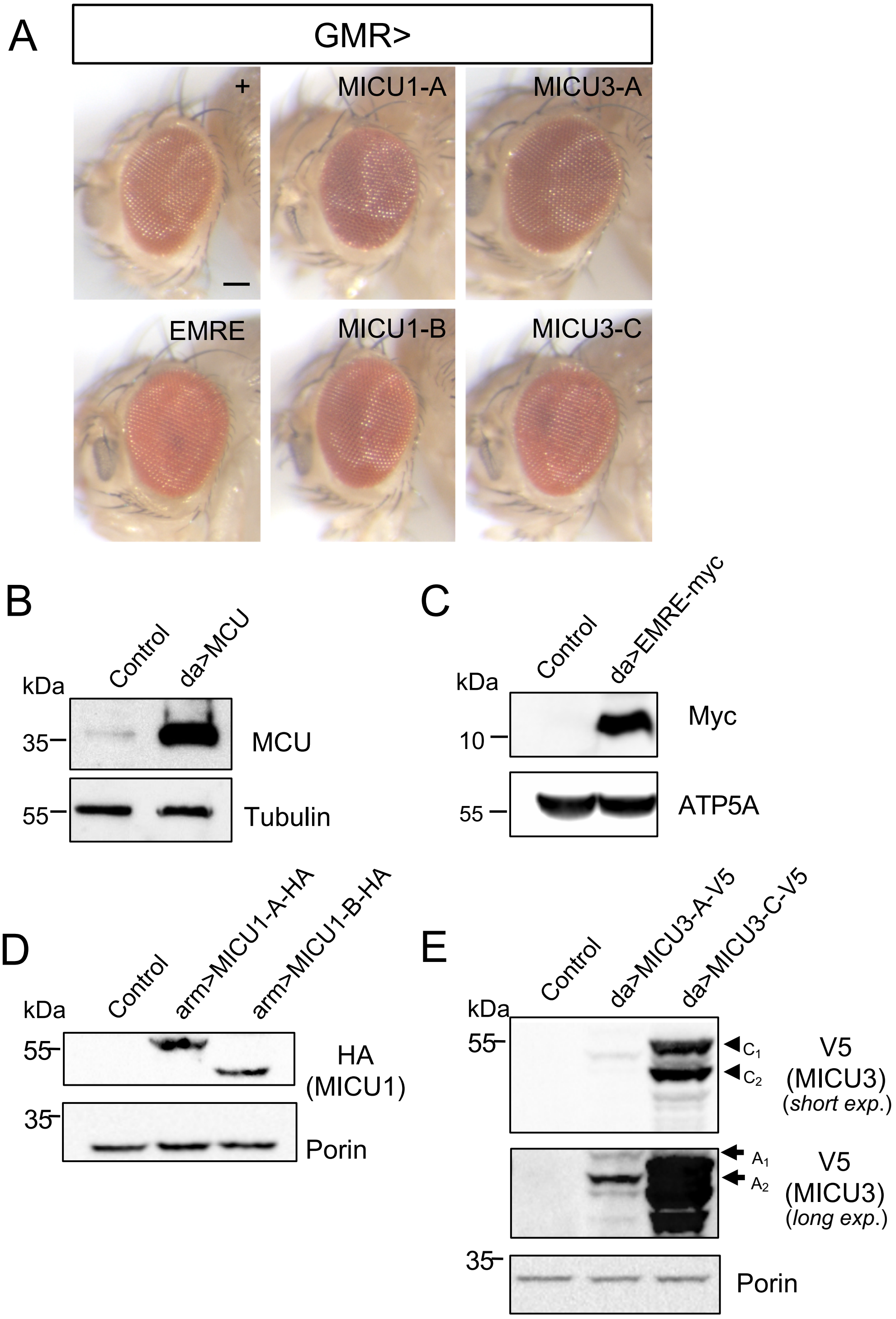

Figure S5. Analysis of transgene expression. Related to Figure 5.

A. Representative images of eye morphology for *GMR-GAL4* driven expression of individual uniporter transgenes. In all conditions the control genotype was the relevant GAL4-only genetic background control (*GAL4*/+). Scale bar = 100 µm.

B. Western blot analysis of ubiquitously (*da-GAL4*) expressed *UAS-MCU* transgene.

C. Western blot analysis of ubiquitously (*da-GAL4*) expressed *UAS-EMRE-myc* transgene.

D. Western blot analysis of transgenic MICU1 expression of *UAS-MICU1-A-3xHA* and *UAS-MICU1-C-HA* lines using a ubiquitous (*arm-GAL4*) driver.

E. Western blot analysis of relative MICU3 expression between *UAS-MICU3-A-V5* and *UAS-MICU3-C-V5* lines using a ubiquitous (*da-GAL4*) driver.

Immunoblots were probed with the indicated antibodies.

Supplementary Table 1 – qPCR Primers (linked to STAR Methods)

REAGENT or RESOURCE	SOURCE	IDENTIFIER
EMRE qPCR Forward Primer: ACATGTCCAGCGTGTACTTTC	This paper	N/A
EMRE qPCR Reverse Primer: GGTATGACGGCACAGAAGATG	This paper	N/A
javelin qPCR Forward Primer: GCGGATTTTTCCGTGAATC	*This paper*	*N/A*
javelin qPCR Reverse Primer: TCTGGCTCTGGGTGTCATC	This paper	N/A
MICU1 qPCR Forward Primer: GTGGCCATGGTCAATCTTTC	This paper	N/A
MICU1 qPCR Reverse Primer: TTGTTGCTGAGTTGGTTGTCA	This paper	N/A
MICU3 qPCR Forward Primer: GATCCACAAACCAAGCGAAT	This paper	N/A
MICU3 qPCR Reverse Primer: CCTCTTCCGGCTCTTGCT	This paper	N/A
RpL32 qPCR Forward Primer: GCCGCTTCAAGGGACAGTATCTG	This paper	N/A
RpL32 qPCR Reverse Primer: AAACGCGGTTCTGCATGAG	This paper	N/A
sulfateless qPCR Forward Primer: AAGCTGTCGATTTGAGTAGCAA	This paper	N/A
sulfateless qPCR Reverse Primer: GACTGTCCACTCGCAATCAG	This paper	N/A
